# The Reaction of Hydrogen Halides with Tetrahydroborate Anion and Hexahydro-*closo*-hexaborate Dianion

**DOI:** 10.3390/molecules26123754

**Published:** 2021-06-20

**Authors:** Igor E. Golub, Oleg A. Filippov, Natalia V. Belkova, Lina M. Epstein, Elena S. Shubina

**Affiliations:** A. N. Nesmeyanov Institute of Organoelement Compounds, Russian Academy of Sciences (INEOS RAS), 28, Vavilova St, 119991 Moscow, Russia; h-bond@ineos.ac.ru (O.A.F.); nataliabelk@ineos.ac.ru (N.V.B.); epst@ineos.ac.ru (L.M.E.)

**Keywords:** borohydride, polyhedral *closo*-borane, proton transfer, Lewis acidity, hydride donor ability, DFT calculations

## Abstract

The mechanism of the consecutive halogenation of the tetrahydroborate anion [BH_4_]^−^ by hydrogen halides (HX, X = F, Cl, Br) and hexahydro-*closo*-hexaborate dianion [B_6_H_6_]^2−^ by HCl via electrophile-induced nucleophilic substitution (EINS) was established by ab initio DFT calculations [M06/6-311++G(d,p) and wB97XD/6-311++G(d,p)] in acetonitrile (MeCN), taking into account non-specific solvent effects (SMD model). Successive substitution of H^−^ by X^−^ resulted in increased electron deficiency of borohydrides and changes in the character of boron atoms from nucleophilic to highly electrophilic. This, in turn, increased the tendency of the B–H bond to transfer a proton rather than a hydride ion. Thus, the regularities established suggested that it should be possible to carry out halogenation more selectively with the targeted synthesis of halogen derivatives with a low degree of substitution, by stabilization of H_2_ complex, or by carrying out a nucleophilic substitution of B–H bonds activated by interaction with Lewis acids (BL_3_).

## 1. Introduction

Halogenated borane derivatives are successfully used as Lewis acids [1,2], as Brønsted superacids [3,4,5], as weakly coordinating anions [6,7], and as components of ionic liquids [8] and solid-state electrolytes [9,10]. Radio-halogenated boron clusters are used in tumor imaging and therapy [11].

Di- and tri-substituted halogenated derivatives of BH_4_^−^ (BH_2_X_2_^−^ and BHX_3_^−^) can be obtained through MBH_4_–MBX_4_ (X = F, Cl) exchange [12,13,14]. However, this reaction always leads to a mixture of products (due to disproportionation and low selectivity of the process), as well as to the formation of by-products as a result of the interaction of substitution products with the initial reagents [12]. Another possible approach to the synthesis of halogenated derivatives is direct halogenation; however, the reported halogenation of BH_4_^−^ by the reaction with HF has resulted in the formation of a mixture of substitution products [14]. However, as exemplified in [15], the use of ^1^H NMR monitoring during the bubbling of *t*BuNH_2_·BH_3_ with anhydrous hydrogen halides HX (X = F, Cl, Br) allows for the reaction to be stopped when the desired halogenation product is formed.

The halogenated derivatives of [B_6_H_6_]^2−^ are typically obtained by the reaction in highly alkaline aqueous solutions in the presence of free halogens X_2_ (X = Cl, Br, I) [16,17]. However, this approach always results in mixtures of several components. Moreover, due to the fast rate of halogenation, the mono- and dihalogen derivatives are available as minor products, while tri- and penta-substituted derivatives are major products. Therefore, the use of preparative scale ion exchange column chromatography is essential for the isolation of pure compounds. However, for geometrical isomers with the same degree of halogenation, the difference in phase mobility is small (5–15%); thus, their separation could be achieved only by repeated ion exchange column chromatography [18].

Due to these factors, there are difficulties in the synthesis of halogen-derived borohydrides. These reactions are not selective; in a general case, a mixture of various substitution products is obtained, and to isolate the desired substitution product, additional separation and purification are necessary. To overcome these difficulties and develop an approach for the targeted synthesis of these compounds, we undertook DFT calculations for the halogenation of tetrahydroborate-anion (BH_4_^−^, which is the smallest available boron hydride model) and hexahydro-*closo*-hexaborate dianion ([B_6_H_6_]^2−^, which is the smallest stable *closo*-borane anion) with hydrogen halides.

Previously, a DFT investigation of halogenation was performed, albeit only for [B_6_H_6_]^2−^ on the example of interaction with HOX (X = Cl, Br) and only for the first two steps [17]. In the case of BH_4_^−^, the DFT study of the stepwise hydrolysis [19] and alcoholysis [20] were reported. Its interaction with strong acids was investigated only for the first reaction step — and only for CF_3_OH [21]. From one side, hydrogen halides (such as HCl and HBr) are strong inorganic acids which violently react with BH_4_^−^ and cause halogenation of *closo*-boranes via the electrophile-induced nucleophilic substitution (EINS) mechanism [22,23]. From the other side, the halogen is the electron-withdrawing group (EWG), which should increase electron deficiency at the boron atom, altering the reactivity of the B–H bond in halogen-substituted derivatives. In this regard, it was interesting to investigate the direct halogenation of BH_4_^−^ and [B_6_H_6_]^2−^ by hydrogen halides.

In the course of our work, we began with the DFT study of the mechanism of direct successive halogenation using the example of the reaction of BH_4_^−^ with HX (X = F, Cl, Br) in acetonitrile (MeCN). Then, using the knowledge gained, we studied halogenation of [B_6_H_6_]^2−^ with HCl in MeCN, which proceeded as an electrophile-induced nucleophilic substitution (EINS) process. Thus, in this work, a principal mechanism of direct halogenation was established for BH_4_^−^ and [B_6_H_6_]^2−^. The changes in the reactivity of the B–H bond entailed by halogens’ introduction were evaluated via calculations of their hydride donor ability (HDA) and proton donor ability (PDA). Hydride donor ability (HDA)—i.e., thermodynamic hydridicity, defined as the Gibbs free energy (∆G°[H^−^]) value for the reaction of hydride ion (H^−^) detachment—is an important reactivity characteristic in transition metals [24,25] and the main group hydrides [20,26,27,28,29]. Similarly, the proton donor ability (PDA), i.e., thermodynamic acidity, was calculated as the Gibbs free energy (∆G°[H^+^]) for the reaction of proton (H^+^) detachment. In the case of anionic boron hydrides, both HDA and PDA parameters have been estimated by only a few researchers [30,31].

## 2. Results and Discussion

### 2.1. Interaction Tetrahydroborate-Anion with Hydrogen Halides

In our previous work [20], we showed (by DFT investigation of stepwise alcoholysis of tetrahydroborate-anion BH_4_^−^) that first proton-hydride transfer is the rate-limiting stage of the reaction, and that the energy profile takes the form of a cascade, since the energy of each next transition state is lower than the one previous. That, in turn, is associated with a decrease in Lewis acidity of the corresponding neutral borane (RO)_x_BH_(3−x)_ and a weakening of B–H bond in [(RO)_x_BH_(4−x)_]^−^ (x = 0−3) anion entailed by the successive substitution of H^−^ by RO^−^ at the boron atom. Thus, the B–H bond activation of BH_4_^−^ and its ability to hydride transfer appeared to be crucial to this process.

It is known that alkaline tetrahydroborates react violently with concentrated mineral acids (such as H_2_SO_4_, HF, etc.) [14,32], yielding the substituted products and diborane, which is formed as a result of interaction between the reaction products. However, as we demonstrated in [30], an introduction of EWG (X^−^, X = F, Cl, Br) to boron atoms increases the Lewis acidity of the parent borane BH_(3−x)_X_x_ and reduces the ability of B–H bonds in [BH_(4−x)_X_x_]^−^ (x = 0−3) to hydride transfer. In this regard, it was interesting to investigate, in the current work, the reaction of BH_4_^−^ with strong mineral acids such as hydrogen halides. For these purposes, we investigated this stepwise process of halogenation of BH_4_^−^ with HX (where X = F, Cl, Br) in acetonitrile (MeCN) at M06/6-311++G(d,p) and wB97XD/6-311++G(d,p) levels of theory.

The dihydrogen-bonded (DHB) complexes are active intermediates of the BH_4_^−^ reaction with HX [33] and HOR [20,21,34,35]. DHB complexes with hydrogen halides HX (X = F, Cl, Br) found in this work (Table S1) were monodentate (except for H_3_BH^−^···HF) with H···H distances of 1.425–1.850 Å and angles ∠X–H···H = 159–178°. Their structures were similar to those found in BH_4_^−^ alcoholysis [20,21], with the geometric parameters typical of DHB complexes [36,37,38]. It should be noted, that for [H_2_XBH]^−^ and [HX_2_BH]^−^ (X = F, Cl, Br) molecules, two coordination modes were found, where halogen atom X was located in *trans*- and *cis*-position to the dihydrogen-bonded HX molecules. The formation energies of DHB complexes with a halogen atom in *trans*-position are slightly less favorable than those of DHB complexes with X in *cis*-position; the difference in the Gibbs free energy of these two complexes, ∆∆G*_f_*°_MeCN_ = ∆G*_f_*°_MeCN_(**INTn^DHB^*_trans_***)– ∆G*_f_*°_MeCN_(**INTn^DHB^*_cis_***), reaches 1.8 kcal/mol. Moreover, the transition states of H_2_ elimination (**TSn^ELIM^** and **TSn^CONC^**, see below) are energetically destabilized when X is located in *trans*-position, compared to those where X is located in *cis*-position. The difference ∆∆G°_MeCN_ ^‡^ = ∆G°_MeCN_ ^‡^(**TSn*_trans_***) − ∆G°_MeCN_ ^‡^(**TSn*_cis_***) can reach up to 2.2 kcal/mol. Thus, herein, we considered only the configurations with X located in *cis*-position to HX.

Upon going from HF to HBr, the enthalpy of DHB complexes’ formation (∆H*_f_*°_MeCN_, Table S1) and other parameters characterizing the H···H interactions strength (E_H···H_ and E^2^ are given in Table S1, and Wiberg bond indices (WBI) and QTAIM delocalization indices (DI)—in Table S3) decreased, in agreement with the HX acidity and hydrogen bond donor strength [39].

**Figure 1:**
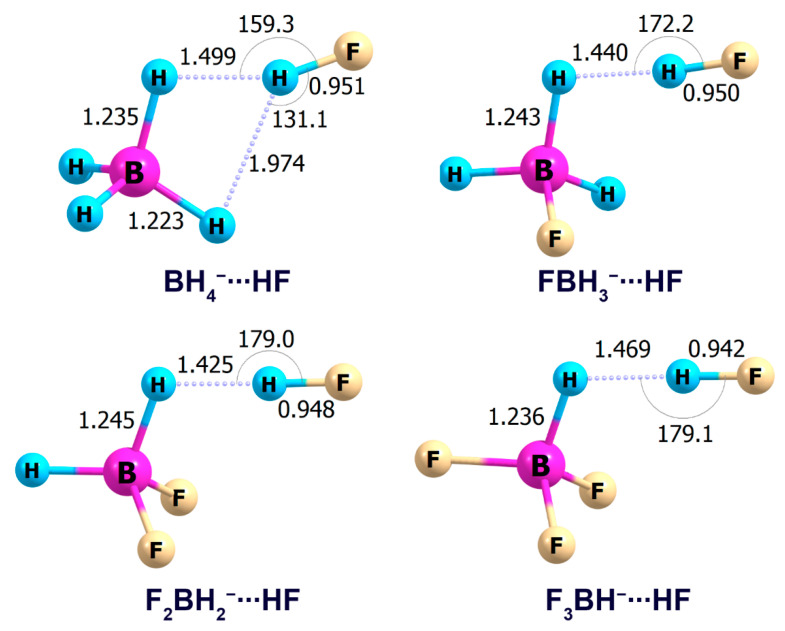
M06-optimized geometries of DHB complexes of [BH_(4−x)_F_x_]^−^ (x = 0–3) with HF (**INTn^DHB^_HF_**). The r_(X···Y)_ bond distances and ∠H···HF angles shown are in Å and °, respectively.

Substitution of one H^−^ by X^−^ (X = F, Cl, Br) in BH_4_^−^ led to an increase of s-character of the B–H bond hybrid orbital in the resulting [BH_3_X]^−^, from 25.0% up to 31.9% (from sp^3^ to sp^2^, Table S3). In general, an increase of s-character of a B–H bond, as in the case of BH_4_^−^ alcoholysis [20], is associated with strengthening of B–H bonds (according to QTAIM delocalization index, DI, Table S4) and weakening of DHB bonds. However, in the case of the BH_4_^−^ reaction with HF for the H_2_FBH^−^···HF and HF_2_BH^−^···HF complexes (Figure 1), there was an increase of DHB interaction strength (E^2^ = 19.2 kcal/mol and 18.6 kcal/mol, respectively, Table S1). In fluoroborohydrides, the strong electron-withdrawing inductive σ-effect—due to the large difference in boron and fluorine electronegativity (∆χ_A-R_ = 2.1) [40]—led to the large positive charge on the boron atom (1.53 for BF_3_) [41]. On the other hand, this effect was compensated by the strong back donation from nonbonding 2p_π_ electron pairs of F to vacant 2p_π_ (B) orbital of the same symmetry [42,43]. For BCl_3_ and BBr_3_, due to a larger size of 2p_π_ orbitals, their effective overlap with vacant boron 2p_π_ orbital decreased; therefore, back-donation for BF_3_ was stronger than for BCl_3_ and BBr_3_, and Lewis acidity decreased in the row BF_3_ > BH_3_ > BCl_3_ > BBr_3_ (as can be seen from their hydride affinity values, HA_298K_, Table S3).

**Figure 2:**
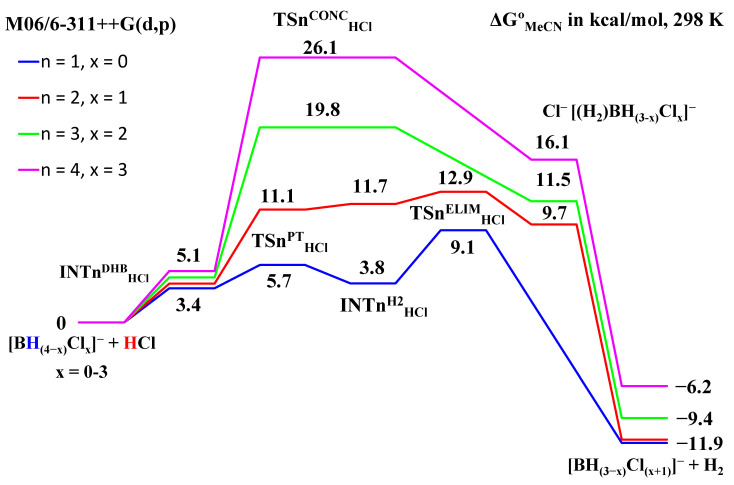
M06-calculated energy profile (∆G°_MeCN_ in kcal/mol) of the stepwise reaction of BH_4_^−^ with HCl in MeCN.

**Figure 3:**
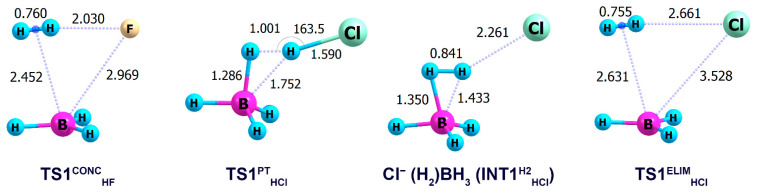
M06-optimized geometries of transition states (TS) and H_2_ reaction intermediates (**INT^H2^**) for the first stage of reaction between BH_4_^−^ with HX (X = F, Cl): concerted transition state of proton-hydride transfer and H_2_ elimination for HF (**TS1^ELIM^_HF_**) and transition states of proton transfer (**TS1^PT^_HCl_**) and H_2_ elimination (**TS1^ELIM^_HCl_**) and H_2_ intermediate (Cl^−^(η^2^-H_2_)BH_3_, **INT1^H2^_HCl_**) for HCl. The r_(X···Y)_ bond distances and ∠H···HF angles shown are in Å and in °, respectively.

The reaction of BH_4_^−^ with HX (where X = F, Cl, Br) in acetonitrile (MeCN) occurs as a successive proton-hydride transfer and H_2_ elimination, yielding [BH_(4−x)_X_x_]^−^ (x = 1−3) halogenated products (Figure 2, Figures S1 and S2). In the case of weak acids like HF (pK_a_^MeCN^_(estim)_ = 25.2 [44]) only one product-like concerted transition state (TS) of proton-hydride transfer and H_2_ elimination was found (**TS1^CONC^_HF_**, Figure 3). In the case of stronger acids HCl (pK_a_^MeCN^= 10.3 [45]) or HBr (pK_a_^MeCN^ = 5.4 [45]), two TS’s were observed: a reagent-like transition state of proton transfer (**TS1^PT^_HCl_**) and a product-like transition state of H_2_ elimination (**TS1^ELIM^_HCl_**). Both concerted TS of proton-hydride transfer/H_2_ elimination **TSn^CONC^_HX_** and TS of H_2_ elimination **TSn^ELIM^_HX_** (preceded by proton transfer step **TSn^PT^_HX_**) exhibited a similar triangle-shaped form of front-side nucleophilic substitution of H_2_ by halide-ion X^−^. The B···H_2_ and H···H distances (Tables S5 and S6) in **TSn^ELIM^_HX_** could be considered indirect indicators of activation barrier height. With the increase of the activation energy, the B···H_2_ distances decreased for the borohydrides with EWG substituents, while at the same time, H···H distances shortened for weak proton donors (such as HF, ROH, Table S7). Similar transition states were described previously in a theoretical study of BH_4_^−^ alcoholysis by weak (MeOH, CF_3_CH_2_OH) and strong (CF_3_OH) proton donors [21].

In the case of strong acids (like HCl, HBr), the formation of a metastable H_2_ intermediate X^−^(η^2^-H_2_)BH_3_ (**INTn^H2^**) was observed (Figure 3). This resembled the intermediates of transition metal hydrides’ protonation [46], which have also been proposed for the protonation of main group element hydrides [21,47,48,49,50,51]. It should be noted that **INTn^H2^** was formed only at the first and second reaction step for HCl, and at the first step for HBr. This was due to an increase in electron deficiency of BH_2_Cl, BH_2_Br, and BHCl_2_ fragments, strengthening of B–H and X···H(H) (X = Cl, Br) bonds, and a decrease of the donation from σ(B–R) (where R = H, Cl, Br) to σ*(H–H–B), which ultimately destabilized the H_2_ intermediate (Table S2). It should be noted that σ(B–R) to σ*(H–H–B) donation from R group in *trans* position to H_2_ complex was the greatest (**E^2^_1_** = 8.8–13.3 kcal/mol) and had a key effect on the stabilization of (η^2^-H_2_) complex. This is also in line with the less energy-demanding attack of HX in the case of [H_2_XBH]^−^ and [HX_2_BH]^−^ bearing X in *trans*-position to HX, in comparison to complexes with X in *cis*-position.

Thus, for the appearance of the transition state of proton transfer, which is absent in the case of weak acids and leads to the (η^2^-H_2_) complex formation, two conditions must apparently be satisfied. First, the pK_a_ of the acid should be greater than the pK_a_ of H_3_B(η^2^-H_2_) and, second, R_3_B borane should be a good Lewis acid; at that, σ(B–R) orbital (such as R = H, SiH_3_, SiF_3_ [51]) should donate to σ*(B–H–H) orbital and R should not be able to π-back-donate to a vacant 2p_π_(B) orbital.

In addition, a strong acid can significantly lower the activation energy of the 2nd reaction stage—that is, the H_2_ elimination. However, the process of H_2_ elimination will still be rate-limiting and will always have the highest value of the activation barrier, because of the relatively high B–H bond strength and its hydride transfer ability. In this regard, the reactivity of substituted borohydrides in reactions involving hydride transfer can be evaluated via their hydride donor ability (HDA_MeCN_, Table S3). In the case of BH_4_^−^ reaction with HF (Figures S1 and S2), the substitution led to HDA_MeCN_ values of [BH_3_F]^−^, [BH_2_F_2_]^−^ and [BHF_3_]^−^, reduced in comparison with BH_4_^−^ (54.64 kcal/mol, 53.12 kcal/mol and 61.08 kcal/mol vs. 64.86 kcal/mol, respectively, for their Li salts; Table S3). As a result, the activation energy decreased by 7.6 and 2.4 kcal/mol for the reaction with the 2nd and 3rd molecule of HF. However, it increased by 6.3 kcal/mol at the last stage (Table 1). Thus, the reaction of BH_4_^−^ with HF followed a trend similar to that of THF·BH_3_ and Me_2_NH·BH_3_ alcoholysis by (CF_3_)_2_CHOH [20,52].

In the case of HCl (Figure 2, Figure S3) and HBr (Figures S4 and S5), the reaction featured two distinct TS’s, i.e., for proton transfer (**TSn^PT^**) and H_2_ elimination (**TSn^ELIM^**). The activation barriers of **TSn^PT^** were small (0.5–4.1 kcal/mol in the case of M06/6-311++G(d,p) and 3.2–7.1 kcal/mol in the case of wB97XD/6-311++G(d,p), see Figures S3 and S5), comparable to the first step of the gas phase reaction between BH_4_^−^ and CF_3_OH (4.2 kcal/mol) [21]. The case of HCl demonstrates how the energy of the complex with molecular hydrogen Cl^−^ [(η^2^-H_2_)BH_(3−x__)_Cl_x_] (x = 0−3) changes during the reaction (Figure 2). At the first step (n = 1, Figure 2), η^2^-H_2_ complex was a local minimum with ∆G°_MeCN_ = 3.8 kcal/mol. Then, at the second step (n = 2, Figure 2), the energy rose, and it became a quasi-stationary intermediate at 11.7 kcal/mol. Over the next two steps, it disappeared, and the transition state became concerted. These changes were not surprising; during the reaction, as H^−^ was replaced by X^−^ (X = Cl, Br), the electron deficiency of the boron atom increased and, on the other hand, π-back-donation from occupied 2p_π_(X) orbital to vacant 2p_π_(B) orbital decreased. This led to an increase in the HDA_MeCN_ of B–H bond in substitution products, and hence to a decrease of the B–H bond’s capability to hydride transfer. As a result, the hydrohalogenation process became concerted, and the activation barriers (∆G°_MeCN_^‡(span)^, Tables S5 and S6) increased (from 9.1 to 26.1 kcal/mol for HCl and from 14.6 to 29.5 kcal/mol for HBr; and, in the case of wB97XD/6-311++G(d,p), from 10.1 to 28.6 kcal/mol for HCl and from 13.1 to 29.2 kcal/mol for HBr). In addition, due to the high electrophilicity of boron atoms in chloro- and bromoborohydrides the propensity of B–H bonds to proton and hydride transfer become comparable in magnitude (PDA^MeCN^ = 85.5 and 83.5 kcal/mol vs. HDA^MeCN^ = 73.0 and 77.6 kcal/mol for Li[BHCl_3_] and Li[BHBr_3_] respectively).

Thus, the value of the activation energy was influenced by two factors: the strength of the acid, which significantly lowered the activation barrier by increasing the polarization of the B–H bond in DHB complexes, and the ability of the occupied 2p_π_(X) orbital to effectively back-donate to vacant 2p_π_(B). In view of both of these factors, hydrogen chloride was the golden reactant, and the observed activation energy values the reaction of BH_4_^−^ with HCl were the lowest (Table 1). Taking into account these findings, the DFT investigation of the reactivity of [B_6_H_6_]^2−^ in electrophile-induced nucleophilic substitution (EINS) was carried out only on an example of the interaction with HCl in acetonitrile (MeCN).

### 2.2. Interaction of Hexahydro-Closo-Hexaborate Dianion with Hydrogen Chloride

**Scheme 1:**
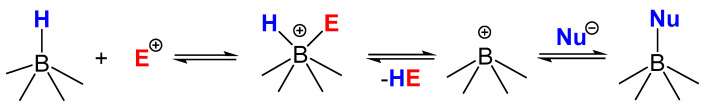
Mechanism of electrophile-induced nucleophilic substitution (EINS).

Polyhedral *closo*-boranes are characterized by rather high values of hydride donor ability (e.g., HDA_MeCN_ = 71.9 kcal/mol for [B_6_H_6_]^2−^ and 79.6 kcal/mol for [B_12_H_12_]^2−^), which indicates the weak ability of the nonactivated B–H bond in *closo*-boranes to hydride transfer [29]. It is not surprising, therefore, that the most common method of functionalization of polyhedral boron hydrides follows the mechanism of electrophile-induced nucleophilic substitution (EINS) (Scheme 1). At the first stage, an electrophile promoter E^+^ is attached to the boron skeleton of a *closo*-borane. Then, the hydride bound to the electrophile is eliminated, with the formation of a quasi-borinium cation [53]. Finally, at the last stage, the nucleophile attachment occurs. At that point, if E^+^ = H^+^, the protonated *closo*-borane intermediate should convert into a η^2^-H_2_ complex—which is generally unstable and readily loses H_2_, making the addition of the nucleophile easier.

However, the main problems of functionalization of polyhedral boron hydrides through this mechanism are low specificity of nucleophilic substitution and difficulties in controlling the degree of substitution [54]. The reactivity of the remaining BH groups in nucleophilic substitution products can be estimated using the hydride donor ability (HDA_MeCN_). Thus, as discussed in this section, applying this approach, we explored the EINS successive mechanism on the example of the reaction of [B_6_H_6_]^2−^ with HCl in acetonitrile.

The [B_6_H_6_]^2−^ is the smallest stable *closo*-borane dianion [29,55,56], where the bulk of electron density is located on the boron atoms of the octahedral skeleton. The high nucleophilicity of boron atoms [57] in [B_6_H_6_]^2−^ leads to its easy protonation. However, an attempt to isolate protonated species from Cs_2_[B_6_H_6_] in acidic aqueous solutions was unsuccessful due to irreversible hydrolysis of [B_6_H_6_]^2−^ to borates and boric acid under these conditions [58]. Nevertheless, [^fac^HB_6_H_6_]^−^ (pK_a_^H2O^ = 7.00) [18,59], in the form of Bu_4_N^+^ and Ph_4_P^+^ salts, was isolated from weakly acidic (pH = 4.5) H_2_O-HCl solutions and characterized by X-ray diffraction [58,60]. It was found that the ^fac^H^+^ proton was located above the B_3_ triangle facets of the octahedron and coordinated through the 4c–-3e bond [18,58,60]. The tendency of [B_6_H_6_]^2−^ to hydrolyze in an acidic environment can be explained by the instability of the hypothetical doubly-protonated B_6_H_8_ *closo*-borane [18,61,62]. However, [B_6_H_6_]^2−^ could be stable in acidic solutions if the protonation were stopped at the stage of [^fac^HB_6_H_6_]^−^ formation [61].

It should be noted that the mechanisms of [B_6_H_6_]^2−^ consecutive halogenation remain practically unexplored. Only the addition of the second halogen to [B_6_H_5_X]^2−^ in the presence of X_2_ and H_2_O (X = Cl, Br) has been explored by DFT, which proceeds with the partial opening of the boron cluster [17].

**Figure 4:**
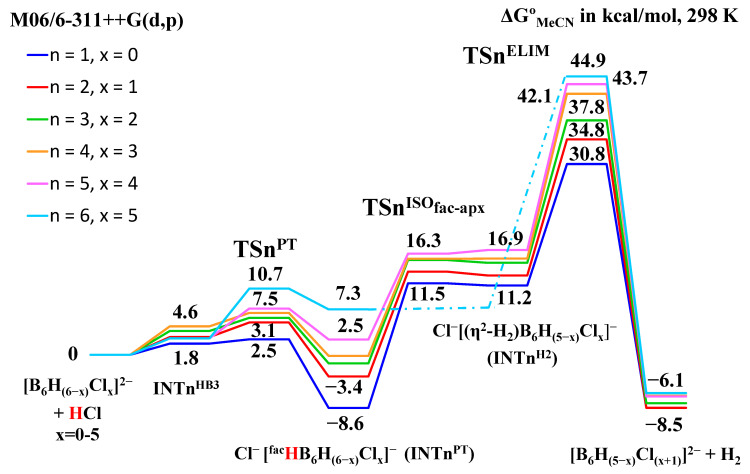
M06-calculated energy profile (∆G°_MeCN_ in kcal/mol) of halogenation of [B_6_H_(6−x)_Cl_x_]^2−^(x = 0−5) by HCl for the pathway: [B_6_H_6_]^2−^ → 1-[B_6_H_5_Cl]^2−^ → 1,2-[B_6_H_4_Cl_2_]^2−^ (*cis*) → 1,2,3-[B_6_H_3_Cl_3_]^2−^ (*fac*) → 1,2,3,4-[B_6_H_2_Cl_4_]^2−^ (*cis*) → 1,2,3,4,6-[B_6_HCl_5_]^2−^.

**Figure 5:**
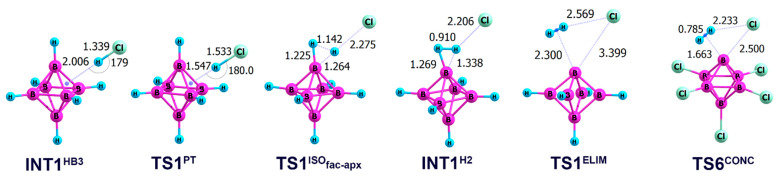
M06-optimized transition states of reaction [B_6_H_6_]^2−^ with HCl. The r_(X···Y)_ bond distances shown are in Å.

Thus, herein, we focused on the investigation of the EINS successive mechanism, simulated on the example of a model reaction in acetonitrile (MeCN) at DFT/M06/6-311++G(d,p) (Figure 4) and wB97XD/6-311++G(d,p) (Figure S7, Table S12) theory levels. It was found that the reaction of [B_6_H_6_]^2−^ with HCl proceeded via preliminary formation of hydrogen-bonded complex on B_3_ triangle facets (**INTn^HB3^**) in three stages (Figure 5). These stages were: (1) protonation of the *closo*-borane cluster (**TSn^PT^**) on one of the B_3_ triangle facets with the formation of protonated form Cl^−^ [^fac^HB_6_H_(6−x)_Cl_x_]^−^ (x = 0–5) (**INTn^PT^**); (2) structural isomerization with H^+^ migration from B_3_ triangle facet to B apex (**TSn^ISOfac-apx^**) with the formation of complexes with molecular hydrogen Cl^−^ [(η^2^-H_2_)B_6_H_(5−x)_Cl_x_]^−^ (η^2^-H_2_-intermediate, **INTn^H2^**); and (3) H_2_ elimination followed by frontal nucleophilic attack on boron atoms by Cl^−^ (**TSn^ELIM^**), which is the last—and rate-limiting—stage. As can be seen (Figure 4), the activation barrier of the protonation stage was very low for all dianions [B_6_H_(6−x)_Cl_x_]^2−^ (x = 0−5) (∆G^‡^ of **TSn^PT^** = 2.5–10.7 kcal/mol); therefore, it did not matter which type of acid we chose to use for protonation. The only important limitation is that the pK_a_ of the proton donor used should be higher than the pK_a_ of Cl^−^ [^fac^HB_6_H_(6−x)_Cl_x_]^−^ (x = 0−5). After protonation, the reaction turned into a classical one—isomerization of Cl^−^ [^fac^HB_6_H_(6−x)_Cl_x_]^−^ to Cl^−^ [(η^2^-H_2_)B_6_H_(5−x)_Cl_x_]^−^ (x = 0–5) (**TSn^ISOfac-apx^**) and H_2_ elimination (**TSn^ELIM^**) in the presence of a nucleophile (Cl^−^). As can be seen from the reaction profile (Figure 4), the H_2_ elimination was the rate-limiting stage and, therefore, the activation barrier of this stage and the selectivity of this process should be determined by the metastable η^2^-H_2_ reaction intermediate.

The first reaction intermediate was the hydrogen-bonded complex (**INTn^HB^^3^**) with HCl coordination on π-density of the B_3_ triangle facet of the [B_6_H_(6−x)_Cl_x_]^2−^ (x = 0–5) polyhedron [63]. It should be noted that the electron density of hydride atoms in [B_6_H_6_]^2−^ is much lower than the electron density of B_3_ triangle facets; therefore, in contrast to BH_4_^−^ [34,35,64], [B_10_H_10_]^2−^ [49,65], and [B_12_H_12_]^2−^ [65], the complexation of proton donors occurred exclusively on B_3_ triangle facets [66,67]. Upon the successive replacement of H^−^ with Cl^−^ in the *closo*-borane structure, gradual increases in the electron deficiency of the skeleton of *closo*-borane and the electrophilicity of boron atoms were observed. As the result, the values of hydride donor ability (HDA_MeCN_, Table S8) increase, whereas the values of proton donor ability (PDA_MeCN_) decrease (Table S8). This indicates a change in the character of boron atoms in *closo*-borane from nucleophilic to highly electrophilic and an increase of the Lewis acidity of the corresponding quasi-borinium cations [B_6_H_(5−x)_Cl_x_]^−^ formed as the result of H^−^ transfer from [B_6_H_(6−x)_Cl_x_]^2−^. Thus, for example, for [B_6_HCl_5_]^2−^, the proton transfer becomes more favorable than hydride transfer, which correlates with better hydrolytic stability of tetra- and penta-halogen derivatives of [B_6_H_6_]^2−^ in acidic aqueous solutions and increased acidity of protonated monosubstituted halogenated derivatives [^fac^HB_6_H_5_X]^−^ (pK_a_^H2O^ for X = Cl, Br, I were 5.35, 5.00 and 4.65, respectively; in comparison, pK_a_^H2O^ = 7.00 for [^fac^HB_6_H_6_]^−^) [42,43]. The study of the electronic structure of polyhedral *closo*-borane dianions [B_6_H_(6−x)_Cl_x_]^2−^ (x = 1−5) utilizing the NBO method showed that, as the substitution proceeded, the polarization of the hydrogen atom occurred. which It became less hydridic (the charge of the hydrogen atom varied from −0.027 to 0.002). These changes were manifested in a weakening of the hydrogen bond (decrease of E_H···B(B2)_ and E^2^, Table S8), an elongation of H···B_3_ distances (Table S9), and a decrease in the enthalpy of H···B_3_ (**INTn^HB^^3^**) complexes’ formation (Table S9).

The energy barrier of the proton transfer reaction was very low (2.5–7.5 kcal/mol); thus, in the presence of acids, the protonated reaction intermediates Cl^−^ [^fac^HB_6_H_(6−x)_Cl_x_]^−^ (x= 0−5) (**INTn^PT^**) were immediately formed (Figure S3). At the early stages of the reaction, these were characterized by significant energy formation (∆G*_f_*°_MeCN_ from −8.5 kcal/mol for Cl^−^ [^fac^HB_6_H_6_]^−^ to −0.4 kcal/mol for Cl^−^ [^fac^HB_6_H_2_Cl_4_]^−^). Previously, instability of [B_6_H_6_]^2−^ in acidic water solutions was explained by possible further protonation and formation of doubly protonated products [18,61,62]. The calculated energy barrier of Cl^−^ [^fac^HB_6_H_(6−x)_Cl_x_]^−^ (x = 0−5) protonation by an equivalent of HCl was higher by 8.8–16.0 kcal/mol (∆G°_MeCN_^‡^ = 9.5–23.0 kcal/mol for **TSn^PT^_2nd_**, Table S10) than protonation of [B_6_H_(6−x)_Cl_x_]^2−^, and its energy increased over the course of halogenation. It should be noted that the formation of doubly-protonated intermediates Cl^−^ (^fac^HB_6_H_(6−x)_Cl_x_^fac^H)·Cl^−^ (x = 0−5) is endoergic (∆G*_f_*°_MeCN_ values ranged from 9.7 kcal/mol for Cl^−^ (^fac^H_2_B_6_H_6_^fac^H)·Cl^−^ to 29.3 kcal/mol for Cl^−^ (^fac^HB_6_HCl_5_^fac^H)·Cl^−^). The activation energy of the reverse reaction (**TS^PT^_2nd(rev)_**, Table S10) was less than 3.8 kcal/mol. All this indicated that it was possible to stop the protonation reaction at the stage of the monoprotonated product, in the presence of equimolar amounts of the proton donor.

It should be noted that the structure of [^fac^HB_6_H_(6−x)_Cl_x_]^−^ (x = 0−5) anions is nonrigid and, in solutions, they are prone to the fluctuation of ^fac^H^+^ proton between octahedral edges [18]. During [B_6_H_6_]^2−^ halogenation, the Cl^−^ [^fac^HB_6_H_(6−x)_Cl_x_]^−^ to Cl^−^ [(η^2^-H_2_)B_6_H_(5−x)_Cl_x_]^−^ (x = 0−5) isomerization (**TSn^ISOfac-apx^**, Figure 5) was observed, with energy barriers decreasing from 20.1 kcal/mol at the first step to 13.8 kcal/mol at the sixth (Table 2)—correlating with an increase in the electrophilic nature of boron atoms during halogenation.

**Scheme 2:**
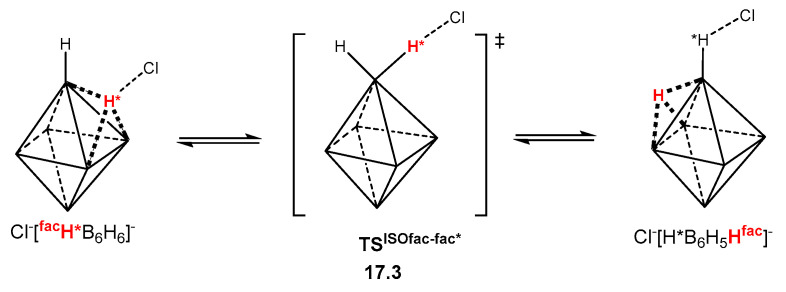
The H^fac^/H^fac^ exchange mechanism in Cl^−^ [^fac^H^*^B_6_H_6_]^−^. ∆G°_MeCN_^‡(span)^ shown in bold numbers are in kcal/mol.

There was also a competitive Cl^−^ [^fac^HB_6_H_(6−x)_Cl_x_]^−^ to [B_6_H_(6−x)_Cl_x_H^fac^*]^−^·Cl^−^ (x = 0−5) isomerization process (TSn^ISOfac-fac*^, Table S9), which corresponded to ^fac^H/H^term^ exchange. As is known, [B_6_H_6_]^2−^ can be completely deuterated in D_2_O solution—and activation energy of the deuteration (18.0 kcal/mol) [18,59] was close to the calculated activation energy of 17.3 kcal/mol for the TSn^ISOfac-fac*^ isomerization of [^fac^HB_6_H_6_]^−^. Thus, the exchange between ^fac^H and H^term^ ligands (TSn^ISOfac-fac*^, Scheme 2) featuring a lower barrier than **TSn^ELIM^** did not result in H_2_ elimination—which requires η^2^-H_2_ complex formation.

A third reaction intermediate, which is Cl^−^ [(η^2^-H_2_)B_6_H_(6−x)_Cl_x_]^−^ complex (**INTn^H2^**) (Table S10) was observed for almost all stages of reaction of [B_6_H_(6−x)_Cl_x_]^2−^ (x = 0−5) with HCl (except [B_6_HCl_5_]^2−^). This complex is a key intermediate of the process which determines the selectivity of nucleophilic substitution, since stabilization of H_2_ at one of the vertices of the *closo*-borane octahedron determines the position of nucleophilic substitution at this vertex. Notably, this complex was not revealed at the gas phase calculations of Cl^−^ [^fac^HB_6_H_6_]^−^ [68]. Similarly to the Cl^−^ [(η^2^-H_2_)B_10_H_9_]^−^ [49] it could be stabilized only in the presence of HX acid residue X^−^.

As the halogenation of [B_6_H_6_]^2−^ progressed, the formation energy of the H_2_ intermediate decreased, and B–H and Cl···H bonds became stronger while H···H interaction weakened. This coincided with a trend toward an increase in the electrophilicity of the B–H bond and an increase in its acidity. Thus, the established regularities allowed for the use of the stabilization of H_2_ complex to control the process of nucleophilic substitution and lower the activation energy of this process.

The same trend was observed for the rate-limiting stage of H_2_ elimination, for which the energy barrier increased from 19.6 to 42.3 kcal/mol (∆G°_MeCN_^‡^(**TSn^PT^**), shown in Table 2, and from 24.6 to 44.6 kcal/mol for wB97XD/6-311++G(d,p), shown in Table S12. It should be noted that nucleophilic substitution by Cl^−^ and solvation contributed greatly to the stabilization of the transition state—which was noticeable, as the activation energies obtained were compared with gas-phase activation energies for H_2_ elimination from [^fac^HB_6_H_6_]^−^ (∆E = 45.9 kcal/mol) [69].

**Scheme 3:**
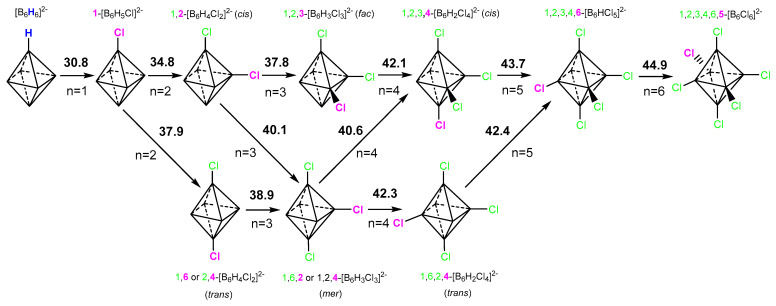
Pathways of [B_6_H_6_]^2−^ halogenation by HCl. Free Gibbs energy span (∆G°_MeCN_^‡(span)^ in kcal/mol) indicated as black bold numbers. H-atoms are omitted for clarity. Cl atoms added at each particular step are shown in magenta.

In the reaction of [B_6_H_6_]^2−^ with HCl there were three branch points at the second, third, and fourth steps (Scheme 3) of halogenation when several geometries of halogenated derivatives were possible (Table 2). However, the optimal reaction pathway (with the lowest activation energy of **TSn^ELIM^** at 2nd and 3rd steps) was [B_6_H_6_]^2−^ → 1-[B_6_H_5_Cl]^2−^ → 1,2-[B_6_H_4_Cl_2_]^2−^ (*cis*) → 1,2,3-[B_6_H_3_Cl_3_]^2−^ (*fac*) → 1,2,3,4-[B_6_H_2_Cl_4_]^2−^ (*cis*) → 1,2,3,4,6-[B_6_HCl_5_]^2−^. The existence of such branch points was found for 1-[B_6_H_5_Cl]^2−^, 1,2-[B_6_H_4_Cl_2_]^2−^ (*cis*) and 1,2,6-[B_6_H_3_Cl_3_]^2−^ (*mer*)-halogenated derivatives, in which there were two types of hydrides with different reactivities (as can be seen from HDA_MeCN_ values, Table S8. As follows from Table 2, the smallest activation barrier corresponded to nucleophilic substitution at the boron atom, the bond of which was characterized by the smallest HDA_MeCN_ value.

It should be noted that under real conditions of halogenation of [B_6_H_6_]^2−^ by X_2_ (X = Cl, Br) in strong alkaline solutions, the reaction is nonspecific. In the second, third, and fourth steps of halogenation, the geometric isomers are formed in different ratios [18]. In this case, for X = I pure geometric isomers, 1,6-[B_6_H_4_I_2_] (*trans*), 1,6,2-[B_6_H_3_I_3_] (*mer*) and 1,6,2,4-[B_6_H_2_I_4_] (*trans*) were observed [70,71]. This was due to the less effective back-donation of larger-sized 2p_π_ iodine orbitals to vacant 2p_π_(B). That, in turn, led to the favorable formation of the protonated complex [^fac^HB_6_H_5_X]^−^, where ^fac^H^+^ was located in *antipodal*-sphere of the [B_6_H_6_]^2−^ octahedron [18,72]. Our calculations showed that, for [^fac^HB_6_H_5_Cl]^−^, the formation energy of the complex with ^fac^H^+^ in the *ipso*-sphere of the [B_6_H_6_]^2−^ octahedron was higher than that of the complex with ^fac^H^+^ in the *antipodal*-sphere (∆∆G*_f_*°_MeCN_ = 3.0 kcal/mol). Moreover, *ipso*-[^fac^HB_6_H_5_Cl]^−^ was the active intermediate of [B_6_H_5_Cl]^2−^ halogenation to 1,2-[B_6_H_4_Cl_2_]^2−^ in our mechanism, as well as in that reported in [17]. Apparently, the larger electron-withdrawing effect of iodine in [B_6_H_(6−x)_I_x_]^2−^ (x = 0–5) derivatives increases the difference between *ipso/antipodal* complexes.

Thus, the above-described reaction mechanism on the example of a model reaction of [B_6_H_6_]^2−^ with HCl suggests the possibility of controlling the selectivity and the depth of the nucleophilic substitution processes by increasing the stabilization of the H_2_ complex on the desired position of the *closo*-borane cluster. Additionally, at the same time, HDA_MeCN_ values for the BH groups would permit the carrying out of a nucleophilic substitution of B–H bonds activated by Lewis acids (BL_3_).

## 3. Materials and Methods

### Computational Details

In the present manuscript, the calculations were performed at the DFT/M06/6-311++G(d,p) level for comparison to the hydride donor ability (HDA) values of boron hydrides and other main group hydrides, as previously calculated by the same method [28,30,73]. Additionally, the values obtained can be used as a reference for assessing the effectiveness of the B–H bond activation by transition metals. In this regard, M06 is a more versatile method than M06-2X (generally recommended for thermochemistry calculations of main group elements) and can be used in cases where multi-reference systems are or might be involved, since it has been parametrized for both main group elements and transition metals [74]. The continuum solute model based on density (SMD) was used for nonspecific solvation, since this model was parametrized to work with the Minnesota functionals family (such as M06, M06-2X, etc.) and has proven to be effective for use in both charged and uncharged systems [75]. The nature of the stationary points on potential energy surfaces was proved by reoptimization of M06 geometries at wB97XD/6-311++G(d,p) level of theory.

All calculations were performed without symmetry constraints using the M06 hybrid functional [74] and wB97XD dispersion-corrected, range-separated hybrid functional [76] implemented in the Gaussian09 (Revision D.01) [77] software package, using 6-311++G(d,p) [78] basis set. Vibrational frequencies were calculated for all optimized complexes at the same level of theory to confirm a character of local minima on the potential energy surface. Visualization-optimized geometries were realized using the Chemcraft 1.8 graphical visualization program [79].

The inclusion of nonspecific solvent effects in the calculations was performed using the SMD method [75]. Acetonitrile (MeCN, ε = 35.7) was chosen as a solvent for optimization because of significant data, specific to MeCN, already existed on reduction potentials, pKa values, and experimental values of hydride donor ability (HDA) of transition metal hydrides [24,25].

Calculations were carried out with an ultrafine integration grid and a very tight SCF option to improve the accuracy of the optimization procedure.

Hydride donor ability (HDA_MeCN_) and proton donor ability (PDA_MeCN_) of E–H bond in MeCN were calculated as Gibbs free energy of hydride or proton transfer:

HDA_MeCN_ = ΔG°[H^−^] _MeCN_ = G°_MeCN_ (E+) + G°_MeCN_ (H−) − G°_MeCN_ (E−H) (1)

PDA_MeCN_ = ΔG°[H^+^] _MeCN_ = G°_MeCN_ (E−) + G°_MeCN_ (H+) − G°_MeCN_ (E−H) (2)

The value of G°_MeCN_ (H^−^) = −413.9 kcal/mol was calculated by M06/6-311++G(d,p) for the H^−^ in MeCN. The values of G°_MeCN_ (H^+^) = −260.2 kcal/mol were taken from [80].

From data obtained during the geometry optimization for each molecule, the most stable configuration was chosen. To find the most stable configuration of cationic boranes, the terminal hydrogen atoms (B–H_term_) were torn off as H^−^ or as H^+^ from each vertex in optimized molecules. In the case of polyhedral *closo*-boranes, due to the rigid frame of the boron cluster [49,50], several quasi-borinium cations were observed, localized on vertices from which hydride was torn off.

The Wiberg bond indices [81] (WBI)—as a measure of electron distribution between two atoms [82]—and natural atomic charges were calculated using the natural-bond orbital (NBO) analysis [83] implemented in GAUSSIAN09 (Revision D.01) [77]. Topological analysis of the electron-density distribution function ρ(r) was performed using the AIMALL [84] program package based on the wave function obtained by M06 calculations. The delocalization index (DI) [85,86,87], a measure of electrons that are shared or exchanged between two atoms or basins obtained from the integration of the Fermi hole density, was directly related to the bond order [87,88]. The X–Y bond ellipticity, ε_X–Y_, was defined as ε = (λ_1_/λ_2_ − 1), where λ_1_ and λ_2_ are the negative eigen values of the Hessian of the electron density at the bond critical point, ordered such that λ_1_ < λ_2_ < 0 [89,90,91]. The energies of H···H or H···B_3_ interactions were calculated as E_H···H_ = 0.5·V(r), according to [92,93].

## 4. Conclusions

The mechanism of the sequential halogenation of tetrahydroborate anions [BH_4_]^−^ by hydrogen halides (HX, X = F, Cl, Br) and halogenation of hexahydro-*closo*-hexaborate dianions [B_6_H_6_]^2−^ was established by ab initio DFT calculations (M06/6-311++G(d,p) and wB97XD/6-311++G(d,p)) in acetonitrile (MeCN), taking into account nonspecific solvent effects (SMD model). The active intermediates of the reaction—XH···HB dihydrogen-bonded (DHB) complexes for [BH_4_]^−^ and ClH···B_3_ complexes for [B_6_H_6_]^2−^—were identified, and their geometric parameters, energetic characteristics, and electron structures were deduced. In the case of [B_6_H_6_]^2−^, an electrophile-induced nucleophilic substitution (EINS) mechanism of halogenation was established. The obtained values of activation energies of H_2_ elimination were relatively high. Nonetheless, the identified pattern in the changes of electronic characteristics, the reactivity of B–H bonds (such as thermodynamic hydridicity and acidity of halogenated products) and the structures of H_2_ complexes suggests that it should be possible to control the degree of halogenation and carry out the targeted synthesis of products with a low degree of substitution using a wide range of synthetic approaches.

## Figures and Tables

**Table 1:** Activation barriers (∆G^‡^_MeCN_, in kcal/mol) for **TSn^CONC^_HX_** (or **TSn^PT^_HX_**/**TSn^ELIM^_HX_**) in the step-wise reaction of BH_4_^−^ with hydrogen halides in MeCN, computed at the M06/6-311++G(d,p) theory level relative to the corresponding DHB complexes.

n	HF	HCl	HBr
	pK_a_^MeCN^ = 25.2 ^a^	pK_a_^MeCN^ = 10.3 ^b^	pK_a_^MeCN^ = 5.4 ^b^
1	27.4	2.3/5.2 ^c^	4.1/6.5 ^c^
2	19.8	0.5/7.3 ^c^	13.0
3	17.5	14.8	21.0
4	23.8	21.0	25.3

^a^ An estimated value is taken from [44]; ^b^ Experimental values are taken from [45]; ^c^ The values for **TSn^PT^_HX_**/**TSn^ELIM^_HX_** are given.

**Table 2:** Hydride donor ability (HDA_MeCN_) of the B–H center in [B_6_H_(6−x)_Cl_x_]^2−^ (x = 0−5), involved in halogenation reactions, and activation barriers (∆G**°_MeCN_**^‡^, in kcal/mol) for TSs for the reaction of [B_6_H_6_]^2−^ with HCl (pK_a_^MeCN^= 10.3 [42]) in MeCN, computed at the M06/6-311++G(d,p) theory level.

n	N of Position	HDA_MeCN_^,a^	∆G°_MeCN_^‡^ (TSn^PT^) ^a^	∆G°_MeCN_^‡^ (TSn^ISOfac-apx^) ^b^	∆G°_MeCN_^‡^ (TSn^ELIM^) ^c^	∆G°_MeCN_^‡(span) d^
1	**1**	71.9	0.7	20.1	19.6	39.3
2	1,**2** (*cis*)	76.6	1.3	19.8	21.9	38.2
	1,**6** (*trans*)	80.0	2.3	19.0	26.0	44.3
3	1,2,**3** (*fac*)	81.0	2.1	19.4	23.0	39.2
	1,2,**6** (*mer*)	84.7	2.2	17.9	26.0	41.5
	1,6,**2** (*mer*)	81.1	3.6	16.7	23.4	40.2
4	1,2,3,**4** (*cis*)	88.6	2.1	17.4	26.6	42.3
	1,6,2,**3** (*cis*)	84.9	4.2	16.0	24.4	42.0
	1,6,2,**4** (*trans*)	88.6	3.9	15.0	26.4	40.2
5	1,2,3,4,**6**	92.5	4.8	13.8	26.8	43.7
	1,6,2,4,**3**	88.4	7.2	37.4 *		42.4
6	1,2,3,4,6,**5**	95.6	8.0	37.6 *		44.9

^a^ HDA^MeCN^ are given for the corresponding reactive B–H centre in [B_6_H_(6−x)_Cl_x_]^2−^ (x = 0−5) molecule; ^b^ Calculated as ∆G°_MeCN_^‡^ (**TSn^PT^**) = G°_MeCN_(**TSn^PT^**) − G°_MeCN_(**INTn^HB^**^3^) ^b^ Calculated as ∆G°_MeCN_^‡^ (**TSn^ISOfac-apx^**) = G°_MeCN_(**TSn^ISOfac-apx^**) − G°_MeCN_(**INTn^PT^**); ^c^ Calculated as ∆G°_MeCN_^‡^(**TSn^ELIM^**) = G°_MeCN_(**TSn^ELIM^**) − G°_MeCN_**(INTn^H2^**); ^d^ Calculated as ∆G°_MeCN_^‡(span)^ = G°_MeCN_(**TSn^ELIM^**) − G°_MeCN_(**INTn^H2^**) for n = 1−4 and as ∆G°_MeCN_^‡(span)^ = G°_MeCN_(**TSn^ELIM^**) − G°_MeCN_([B_6_H_(6−x)_Cl_x_]^2−^) − G°_MeCN_(HCl) for n = 5−6; * **TSn^CONC^** for 5th and 6th halogenation steps.

## Data Availability

Not applicable.

## Supplementary Materials

The following are available online, Table S1. M06-computed geometric parameters (distances r_(X–Y)_ in Å, angles in °) of dihydrogen-bonded (DHB) complexes (INTn^DHB^) of [BH_(4−x)_X_x_] − (x = 0–3) with HX (X = F, Cl, Br) and their energies. Table S2. M06-computed geometric parameters (distances r_(X–Y)_ in Å, angles in °) of H_2_ complexes of X^−^[(H_2_)BH_(4−x)_X_x_]− (x = 1–2, X = Cl, Br) (INT^H2^) and their energies. Table S3. Hydride affinity (HA_298K_ in kcal/mol) of [BH_(4−x)_X_x_]^−^ (x = 0–3) (X = F, Cl, Br). Hybridization of boron orbitals in B–H moieties, QTAIM delocalization indices (DI) of BH bond in of [BH_(4−x)_X_x_]^−^ (x = 0–3, X = F, Cl, Br). Hydride (HDA_MeCN_ in kcal/mol) and proton (PDA_MeCN_ in kcal/mol) donor abilities for Li[BH_(4−x)_X_x_] in MeCN. Table S4. QTAIM delocalization indices (DI) and Wiberg bond indices (WBI) for H···H contacts, their differences for XH and HB bonds, and hybridization of boron orbitals in BH moieties of M06-computed geometries of DHB complexes for [BH_(4−x)_X_x_]^−^ (x = 0–3) with HX (X = F, Cl, Br) (INTn^DHB^). Table S5. Free Gibbs activation energy in acetonitrile (∆G°_MeCN_^‡^ in kcal/mol) of the transition state of H_2_ elimination (TSn^ELIM^_HX_), and concerted transition state of proton-hydride transfer/H_2_ elimination (TSn^CONC^_HX_) for the reaction of BH_4_^−^ with HX (X = F, Cl, Br)—and their M06-optimized geometric parameters. Table S6. Free Gibbs activation energy in acetonitrile (∆G°_MeCN_^‡^ in kcal/mol) of the transition state of H_2_ elimination (TSn^ELIM^_HX_) and concerted transition state of proton-hydride transfer/H_2_ elimination (TSn^CONC^_HX_) for the reaction of BH_4_^−^ with HX (X = F, Cl, Br) and their wB97XD-optimized geometric parameters. Table S7. Free Gibbs activation energy in dichloromethane (∆G°_DCM_^‡^ in kcal/mol) of the concerted transition state of proton-hydride transfer/H_2_ elimination (TS^CONC^_ROH_) for the reaction of BH_4_^−^ with HOR [ROH = MeOH, CF_3_CH_2_OH (TFE), (CF_3_)_2_CHOH (HFIP)] and their M06-optimized geometric parameters. Table S8. Hydride (HDA_MeCN_ in kcal/mol) and proton (PDA_MeCN_ in kcal/mol) donor abilities for [B_6_H_(6−x)_Cl_x_]^2−^ (x = 0−5) in MeCN. Table S9. M06-computed geometric (distances r_(X–Y)_ in Å, angles in degrees) parameters of hydrogen-bonded complexes of [B_6_H_(6−x)_Cl_x_]^2−^ (x = 0–5) with HCl (INTn^HB^) and their energies. Table S10. M06-computed geometric (distances r_(X–Y)_ in Å, angles in degrees) parameters of hydrogen-bonded complexes of Cl^−^ [^fac^HB_6_H_(6−x)_Cl_x_]^−^ (x = 0–5) with HCl and their energies. Table S11. M06-computed geometric parameters (distances r_(X–Y)_ in Å, angles in °) of H_2_ complexes of Cl^−^[(H_2_)B_6_H_(5−x)_Cl_x_]^−^ (n = 0–5) (INTn^H2^) and their energies. Table S12. Activation barriers (∆G°_MeCN_^‡^, in kcal/mol) for TSs for the reaction of [B_6_H_6_]^2−^ with HCl (pK_a_^MeCN^ = 10.3) in MeCN, computed at the wB97XD/6-311++G(d,p) theory level. Figure S1. M06-calculated energy profile (∆G°_MeCN_ in kcal/mol) of the reaction of BH_4_^−^ with HF in MeCN. Figure S2. wB97XD-calculated energy profile (∆G°_MeCN_ in kcal/mol) of the reaction of BH_4_^−^ with HF in MeCN. Figure S3. wB97XD-calculated energy profile (∆G°_MeCN_ in kcal/mol) of the reaction of BH_4_^−^ with HCl in MeCN. Figure S4. M06-calculated energy profile (∆G°_MeCN_ in kcal/mol) of the reaction of BH_4_^−^ with HBr in MeCN. Figure S5. wB97XD-calculated energy profile (∆G°_MeCN_ in kcal/mol) of the reaction of BH_4_^−^ with HBr in MeCN. Figure S6. M06-optimized geometries of the protonated forms [^fac^HB_6_H_(__6−x)_Cl_x_]^−^ (n = 0−4) and their free Gibbs formation energies (∆G*_f_*°^MeCN^ in kcal/mol). Figure S7. wB97XD-calculated energy profile of halogenation of [B_6_H_(6−x)_Cl_x_]^2−^ (x = 0−5) by HCl for the pathway: [B_6_H_6_]^2−^ → 1-[B_6_H_5_Cl]^2−^ → 1,2-[B_6_H_4_Cl_2_]^2−^ (*cis*) → 1,2,3-[B_6_H_3_Cl_3_]^2−^ (fac) → 1,2,3,4-[B_6_H_2_Cl_4_]^2−^ (*cis*) → 1,2,3,4,6-[B_6_HCl_5_]^2−^. Animation of the vibration motion at the imaginary frequency for the TSs (GIF). Cartesian coordinates and electronic energies (in Hartrees) for the calculated species (XYZ).

## Sample Availability

Not available.
