# Supplementary material for: The Reaction of Hydrogen Halides with Tetrahydroborate Anion and Hexahydro-closo-hexaborate Dianion
Source: Molecules. 2021 Jun 20;26(12):3754. doi: 10.3390/molecules26123754 (PMC8235096; doi:10.3390/molecules26123754)
Supplement: Supplementary file 1 [file molecules-26-03754-s001.zip › molecules-1220861-supplementary/BH4_B6H6_SI_final_REF.pdf]

Reaction of Hydrogen Halides with Tetrahydroborate Anion and Hexahydro-*closo*-hexaborate Dianion

Igor E. Golub*, Oleg A. Filippov, Natalia V. Belkova, Lina M. Epstein, and Elena S. Shubina*

A. N. Nesmeyanov Institute of Organoelement Compounds, Russian Academy of Sciences (INEOS RAS), 28 Vavilova St, 119991 Moscow, Russia; h-bond@ineos.ac.ru (O.A.F.); nataliabelk@ineos.ac.ru (N.V.B.); epst@ineos.ac.ru (L.M.E.)

* Correspondence: seraph347@gmail.com (I.E.G.); shu@ineos.ac.ru (E.S.S.)

Supplementary Materials

Table S1. M06-computed geometric parameters (distances $r_{(X–Y)}$ in Å, angles in °) of dihydrogen-bonded (DHB) complexes (INTn^{DHB}) of $[BH_{(4–x)}X_x]^–$ (x = 0–3) with HX (X = F, Cl, Br), their formation energy ($\Delta G_f°_{MeCN}$ and $\Delta H_f°_{MeCN}$ in kcal/mol), electron densities in (+3;−1) critical point of H···H bond (ρ_c in au), Laplacian of electron density at critical point ($\nabla^2\rho_c$ in au), bond ellipticity (ε), energy of H···H interaction ($E_{H\cdots H}$ = 0.5 · V(r)[1, 2] in kcal/mol) and donation energy (E^2 in kcal/mol) from σ(B–H) to σ*(H–X) estimated from 2nd-order perturbative analysis of donor acceptor interactions within natural-bond orbital (NBO) analysis. **S3**

Table S2. M06-computed geometric parameters (distances $r_{(X–Y)}$ in Å, angles in °) of H_2 complexes of $X^-[(H_2)BH_{(4–x)}X_x]^–$ (n = 1–2, X= Cl, Br) (INTn^{H2}), their formation energy ($\Delta G_f°_{MeCN}$ and $\Delta H_f°_{MeCN}$ in kcal/mol), donation energy (E_2 in kcal/mol) from σ(B–H/X) to σ*(H–H–B) (**E^2_1**), from σ(H–H–B) to σ*(B–H/X) (**E^2_2**) and from p(X) to σ*(H–H–B) (**E^2_3**) estimated from 2nd-order perturbative analysis of donor acceptor interactions within natural-bond orbital (NBO) analysis, QTAIM energy of H_2···H, H···H and B···H interactions ($E_{X\cdots Y}$ = 0.5 · V(r)[1, 2] in kcal/mol). **S3**

Table S3. Hydride affinity (HA_{298K} in kcal/mol) of $[BH_{(4–x)}X_x]^-$ (x = 0–3) (X = F, Cl, Br). Hybridization of boron orbitals in B–H moieties, QTAIM delocalization indices (DI) of BH bond in of $[BH_{(4–x)}X_x]^-$ (X = F, Cl, Br). Hydride (HDA_{MeCN} in kcal/mol) and proton (PDA_{MeCN} in kcal/mol) donor abilities for $Li[BH_{(4-x)}X_x]$ in MeCN. S4

Table S4. QTAIM delocalization indices (DI) and Wiberg bond indices (WBI) for H···H contacts, their differences for XH and HB bond and hybridization of boron orbitals in BH moieties of M06-computed geometries of DHB complexes for $[BH_{(4–x)}X_x]^-$ (x = 0–3) with HX (X = F, Cl, Br) (INTn^{DHB}). S4

Figure S1. M06-calculated energy profile ($\Delta G°_{MeCN}$ in kcal/mol) of the reaction of BH_4^- with HF in MeCN. S5

Figure S2. wB97XD-calculated energy profile ($\Delta G°_{MeCN}$ in kcal/mol) of the reaction of BH_4^- with HF in MeCN. S5

Figure S3. wB97XD-calculated energy profile ($\Delta G°_{MeCN}$ in kcal/mol) of the reaction of BH_4^- with HCl in MeCN. S6

Figure S4. M06-calculated energy profile ($\Delta G°_{MeCN}$ in kcal/mol) of the reaction of BH_4^- with HBr in MeCN. S7

Figure S5. wB97XD-calculated energy profile ($\Delta G°_{MeCN}$ in kcal/mol) of the reaction of BH_4^- with HBr in MeCN. S7

Table S5. Free Gibbs activation energy in acetonitrile ($\Delta G°_{MeCN}{}^‡$ in kcal/mol) of the transition state of H_2 elimination ($TSn^{ELIM}{}_{HX}$) and concerted transition state of proton-hydride transfer/H_2 elimination ($TSn^{CONC}{}_{HX}$) for reaction of BH_4^- with HX (X = F, Cl, Br) and their M06-optimized geometric parameters. S8

Table S6. Free Gibbs activation energy in acetonitrile ($\Delta G°_{MeCN}{}^‡$ in kcal/mol) of the transition state of H_2 elimination ($TSn^{ELIM}{}_{HX}$) and concerted transition state of proton-hydride transfer/H_2 elimination ($TSn^{CONC}{}_{HX}$) for reaction of BH_4^- with HX (X = F, Cl, Br) and their wB97XD-optimized geometric parameters. S8

Table S7. Free Gibbs activation energy in dichloromethane ($\Delta G°_{DCM}{}^{‡}$ in kcal/mol)[5] of the concerted transition state of proton-hydride transfer/H_2 elimination (TS^{CONC}_{ROH}) for reaction of BH_4^- with HOR [ROH = MeOH, CF_3CH_2OH (TFE), $(CF_3)_2CHOH$ (HFIP)] and their M06-optimized geometric parameters. S10

Table S8. Hydride (HDA_{MeCN} in kcal/mol) and proton (PDA_{MeCN} in kcal/mol) donor abilities for $[B_6H_{(6-x)}Cl_x]^{2-}$ (x = 0-5) in MeCN. S10

Table S9. M06-computed geometric (distances $r_{(X-Y)}$ in Å, angles in degrees) parameters of hydrogen-bonded complexes of $[B_6H_{(6-x)}Cl_x]^{2-}$ (x = 0–5) with HCl ($INTn^{HB}$), their formation energy ($\Delta G_f°_{MeCN}$ and $\Delta H_f°_{MeCN}$ in kcal/mol), electron densities at the H···B(B2) bond (+3;−1) critical point (ρ_c in au), Laplacian of electron density at critical point ($\nabla^2\rho_c$ in au), bond ellipticity (ε) and energy of H···B(B2) interaction ($E_{H\cdots B(B2)} = 0.5 \cdot V(r)$ [1, 2] in kcal/mol) and donation energy (E^2_{HB} in kcal/mol) from σ(B–B–B) to σ*(H–Cl) estimated from 2nd-order perturbative analysis of donor acceptor interactions within natural-bond orbital (NBO) analysis. S11

Table S10. M06-computed geometric (distances $r_{(X-Y)}$ in Å, angles in degrees) parameters of hydrogen bonded complexes of $Cl^-\cdot[^{fac}HB_6H_{(6-x)}Cl_x]^-$ (x = 0–5) with HCl, their formation energy ($\Delta G_f°_{MeCN}$ and $\Delta H_f°_{MeCN}$ in kcal/mol), electron densities at the H···B(B2) bond (+3;−1) critical point (ρ_c in au), Laplacian of electron density at critical point ($\nabla^2\rho_c$ in au), bond ellipticity (ε) and energy of H···B(B2) interaction ($E_{H\cdots B(B2)} = 0.5 \cdot V(r)$ [1, 2] in kcal/mol). S11

Table S11. M06-computed geometric parameters (distances $r_{(X-Y)}$ in Å, angles in °) of H_2 complexes of $Cl^-[(H_2)B_6H_{(5-x)}Cl_x]^-$ (x = 0–5) ($INTn^{H2}$), their formation energy ($\Delta G_f°_{MeCN}$ and $\Delta H_f°_{MeCN}$ in kcal/mol), electron densities in (+3;−1) donation energy (E^2 in kcal/mol) from σ(B–H/Cl) to σ*(H–H–B) (**E^2_1**), from σ(H–H–B) to σ*(B–H/Cl) (**E^2_2**) and from p(Cl) to σ*(H–H–B) (**E^2_3**) estimated from 2nd-order perturbative analysis of donor acceptor interactions within natural-bond orbital (NBO) analysis. S12

Figure S6. M06-optimized geometries of the protonated forms $[^{fac}HB_6H_{(6-x)}Cl_x]^-$ (x = 0–4) and their free Gibbs formation energies ($\Delta G_f°_{MeCN}$ in kcal/mol). S13

Figure S7. wB97XD-calculated energy profile of halogenation of $[B_6H_{(6-x)}Cl_x]^{2-}$(x = 0–5) by HCl for the pathway: $[B_6H_6]^{2-}$ → 1-$[B_6H_5Cl]^{2-}$ → 1,2-$[B_6H_4Cl_2]^{2-}$ (*cis*) → 1,2,3-$[B_6H_3Cl_3]^{2-}$ (*fac*) → 1,2,3,4-$[B_6H_2Cl_4]^{2-}$ (*cis*) → 1,2,3,4,6-$[B_6HCl_5]^{2-}$. S14

Table S12. Activation barriers ($\Delta G°_{MeCN}{}^{‡}$, in kcal/mol) for TSs for the reaction of $[B_{6x}H_6]^{2-}$ with HCl (pK_a^{MeCN}= 10.3) in MeCN computed at the wB97XD/6-311++G(d,p) theory level. S15

References S16

Table S1. M06-computed geometric parameters (distances $r_{(X–Y)}$ in Å, angles in °) of dihydrogen-bonded (DHB) complexes (**INT^{DHB}**) of $[BH_{(4-x)}X_x]^-$ (x = 0–3) with HX (X = F, Cl, Br), their formation energy ($\Delta G_f^o{}_{MeCN}$ and $\Delta H_f^o{}_{MeCN}$ in kcal/mol), electron densities in (+3;–1) critical point of H···H bond (ϱ_c in au), Laplacian of electron density at critical point ($\nabla^2\varrho_c$ in au), bond ellipticity (ε), energy of H···H interaction ($E_{H\cdots H}$ = 0.5·V(r) [1, 2] in kcal/mol) and donation energy (E^2 in kcal/mol) from σ(B–H) to σ*(H–X) estimated from 2nd-order perturbative analysis of donor acceptor interactions within natural-bond orbital (NBO) analysis.

DHB complex	$r_{(H_X\cdots H_B)}$	$r_{(XH)}$	$\Delta r_{(XH)}$	$\angle_{XH\cdots H}$	$r_{(HB)}$	$\Delta r_{(HB)}$	$\angle_{H\cdots HB}$	$\Delta G_f^o{}_{MeCN}$	$\Delta H_f^o{}_{MeCN}$	ϱ_c	$\nabla^2\varrho_c$	ε	$E_{H\cdots H}$	E^2
$H_3BH^-\cdots HF$	1.499<br>1.974	0.951	0.027	159<br>131	1.235<br>1.223	−0.002<br>−0.014	100<br>79	2.7	−4.9	0.036	0.072	0.25	−8.0	16.1<br>2.8
$FH_2BH^-\cdots HF$	1.440	0.950	0.026	172	1.243	0.005	105	2.6	−4.3	0.039	0.074	0.13	−9.2	19.2
$F_2HBH^-\cdots HF$	1.425	0.948	0.024	179	1.245	0.008	111	4.5	−3.7	0.039	0.082	0.08	−9.2	18.6
$F_3BH^-\cdots HF$	1.469	0.942	0.018	179	1.236	−0.001	113	5.1	−2.5	0.034	0.075	0.08	−7.6	15.7
$H_3BH^-\cdots HCl$	1.580	1.324	0.031	172	1.236	−0.001	103	3.4	−4.2	0.031	0.064	0.13	−5.6	15.5
$ClH_2BH^-\cdots HCl$	1.641	1.315	0.021	171	1.220	−0.017	106	3.8	−3.7	0.026	0.061	0.16	−4.4	10.6
$Cl_2HBH^-\cdots HCl$	1.656	1.308	0.014	174	1.209	−0.028	117	4.4	−3.4	0.023	0.058	0.10	−3.7	7.3
$Cl_3BH^-\cdots HCl$	1.715	1.305	0.011	178	1.201	−0.037	118	5.1	−2.5	0.020	0.052	0.09	−3.0	5.0
$H_3BH^-\cdots HBr$	1.726	1.452	0.024	169	1.234	−0.004	103	3.6	−3.7	0.023	0.053	0.15	−3.5	9.5
$BrH_2BH^-\cdots HBr$	1.797	1.444	0.017	165	1.217	−0.020	104	3.9	−3.6	0.020	0.048	0.19	−2.9	6.2
$Br_2HBH^-\cdots HBr$	1.840	1.439	0.011	160	1.204	−0.033	113	4.4	−3.0	0.017	0.043	0.13	−2.4	3.6
$Br_3BH^-\cdots HBr$	1.850	1.437	0.009	163	1.196	−0.041	115	4.2	−3.0	0.016	0.041	0.02	−2.2	3.0

Table S2. M06-computed geometric parameters (distances $r_{(X–Y)}$ in Å, angles in °) of η^2-H_2 complexes of $X^-[(H_2)BH_{(4-x)}X_x]^-$ (x = 1–2, X= Cl, Br) (**INTn^{H2}**), their formation energy ($\Delta G_f^o{}_{MeCN}$ and $\Delta H_f^o{}_{MeCN}$ in kcal/mol), donation energy (E_2 in kcal/mol) from σ(B–H/X) to σ*(H–H–B) (**$E^2{}_1$**), from σ(H–H–B) to σ*(B–H/X) (**$E^2{}_2$**) and from p(X) to σ*(H–H–B) (**$E^2{}_3$**) estimated from 2nd-order perturbative analysis of donor acceptor interactions within natural-bond orbital (NBO) analysis, QTAIM energy of $H_2\cdots H$, H···H and B···H interactions ($E_{X\cdots Y}$ = 0.5·V(r) [1, 2] in kcal/mol).

η^2-H_2 complex	$r_{(H\cdots H)}$	$r_{(B\cdots H1)}$	$\Delta r_{(B\cdots H1)}$	$r_{(B\cdots H_2)}$	$r_{(B\cdots H2)}$	$\Delta r_{(B\cdots H2)}$	$r_{(H2\cdots X)}$	$\Delta r_{(H2\cdots X)}$	$\Delta G_f^o{}_{MeCN}$	$\Delta H_f^o{}_{MeCN}$	$E^2{}_1$	$E^2{}_2$	$E^2{}_3$	$E_{H2\cdots X}$	$E_{H\cdots H}$	$E_{B\cdots H}$
$Cl^-\cdot(H_2)BH_3$	0.841	1.351	0.124	1.327	1.432	0.205	2.272	0.978	3.8	−3.7	13.3<br>2.9<br>2.3	3.7<br>1.1<br>1.3	15.7<br>1.2	−3.9	−63.8	−64.9
$Br^-\cdot(H_2)BH_3$	0.846	1.343	0.116	1.340	1.464	0.237	2.302	1.008	8.0	0.2	12.0<br>2.1<br>2.0	4.2<br>1.4<br>1.4	23.7<br>2.1	−4.6	−63.1	−64.4
$Cl^-\cdot(H_2)BH_2Cl$	0.853	1.342	0.115	1.363	1.508	0.281	2.042	0.748	11.7	3.0	8.8<br>7.9<br>4.6	11.5<br>5.1<br>3.3	38.1<br>3.3<br>1.5	−8.0	−61.7	−63.3

Table S3. Hydride affinity (HA_{298K} in kcal/mol) of $[BH_{(4-x)}X_x]^-$ (x = 0–3) (X = F, Cl, Br). Hybridization of boron orbitals in B–H moieties, QTAIM delocalization indices (DI) of BH bond in $[BH_{(4-x)}X_x]^-$ (X = F, Cl, Br). Hydride (HDA_{MeCN} in kcal/mol) and proton (PDA_{MeCN} in kcal/mol) donor abilities for $Li[BH_{(4-x)}X_x]$ in MeCN.

Molecule	HA_{298K}[a]	Molecule	(sp^N) B–H	DI (BH)	Molecule	HDA_{MeCN}[b]	PDA_{MeCN}
BH_3	73.7	BH_4^-	3.0	0.537	$LiBH_4$	64.86	96.51
FBH_2	63.2	$[BH_3F]^-$	2.7	0.503	$Li[BH_3F]$	54.64	104.37
F_2BH	60.0	$[BH_2F_2]^-$	2.5	0.468	$Li[BH_2F_2]$	53.12	107.48
F_3B	70.5	$[F_3BH]^-$	2.1	0.442	$Li[BHF_3]$	61.08	101.22
$ClBH_2$	80.9	$[BH_3Cl]^-$	2.6	0.544	$Li[BH_3Cl]$	65.34	88.23
Cl_2BH	88.1	$[BH_2Cl_2]^-$	2.4	0.548	$Li[BH_2Cl_2]$	69.80	93.85
Cl_3B	95.8	$[BHCl_3]^-$	2.2	0.552	$Li[BHCl_3]$	73.02	85.49
$BrBH_2$	87.3	$[BH_3Br]^-$	2.6	0.553	$Li[BH_3Br]$	69.47	88.97
Br_2BH	97.3	$[BH_2Br_2]^-$	2.4	0.571	$Li[BH_2Br_2]$	74.67	88.81
Br_3B	104.9	$[BHBr_3]^-$	2.0	0.592	$Li[BHBr_3]$	77.60	83.47

[a] Calculated values are taken from [3]. [b] Calculated values are taken from [4].

Table S4. QTAIM delocalization indices (DI) and Wiberg bond indices (WBI) for H···H contacts, their differences for XH and HB bond and hybridization of boron orbitals in BH moieties of M06-computed geometries of DHB complexes for $[BH_{(4-x)}X_x]^-$ (x = 0–3) with HX (X = F, Cl, Br) (**INT**^{DHB}).

DHB complex	$DI_{(XH\cdots HB)}$	$\Delta DI_{(XH)}$	$\Delta DI_{(HB)}$	$WBI_{(XH\cdots HB)}$	$\Delta WBI_{(XH)}$	$\Delta WBI_{(HB)}$	H–B(sp^N)
$H_3BH^-\cdots HF$	0.082	−0.060	−0.046	0.078	−0.164	−0.098	3.2
	0.022		−0.009	0.009		−0.010	3.0
$FH_2BH^-\cdots HF$	0.095	−0.057	−0.053	0.105	−0.142	−0.133	3.0
$F_2HBH^-\cdots HF$	0.097	−0.052	−0.052	0.057	−0.120	−0.143	2.7
$F_3BH^-\cdots HF$	0.087	−0.045	−0.046	0.102	−0.093	−0.124	2.3
$H_3BH^-\cdots HCl$	0.111	−0.161	−0.047	0.079	−0.161	−0.119	3.2
$ClH_2BH^-\cdots HCl$	0.094	−0.131	−0.038	0.057	−0.110	−0.087	2.8
$Cl_2HBH^-\cdots HCl$	0.084	−0.104	−0.036	0.044	−0.068	−0.071	2.5
$Cl_3BH^-\cdots HCl$	0.074	−0.083	−0.032	0.033	−0.046	−0.051	2.3
$H_3BH^-\cdots HBr$	0.094	−0.145	−0.034	0.049	−0.124	−0.080	3.1
$BrH_2BH^-\cdots HBr$	0.077	−0.122	−0.028	0.032	−0.087	−0.055	2.7
$Br_2HBH^-\cdots HBr$	0.064	−0.100	−0.024	0.021	−0.054	−0.039	2.5
$Br_3BH^-\cdots HBr$	0.063	−0.089	−0.025	0.018	−0.047	−0.033	2.3

Figure S1. M06-calculated energy profile ($\Delta G°_{MeCN}$ in kcal/mol) of the reaction of BH_4^- with HF in MeCN.

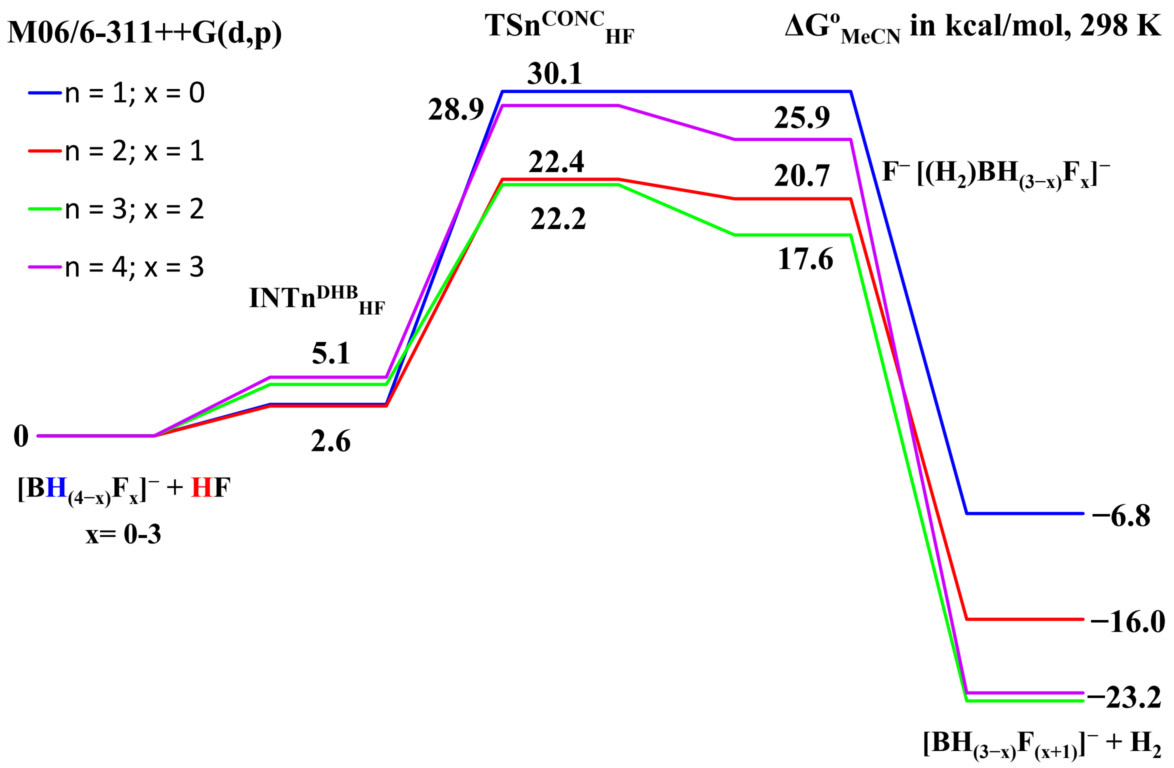

Figure S2. wB97XD-calculated energy profile ($\Delta G°_{MeCN}$ in kcal/mol) of the reaction of BH_4^- with HF in MeCN.

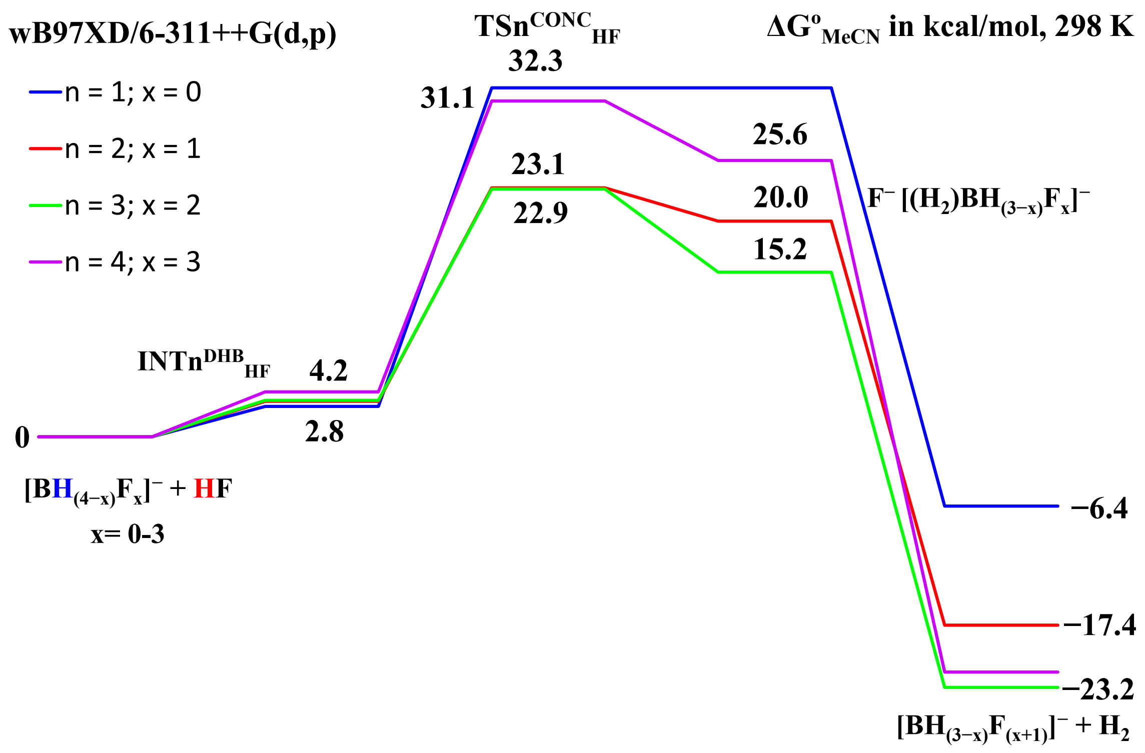

Figure S3. wB97XD-calculated energy profile ($\Delta G°_{MeCN}$ in kcal/mol) of the reaction of BH_4^- with HCl in MeCN.

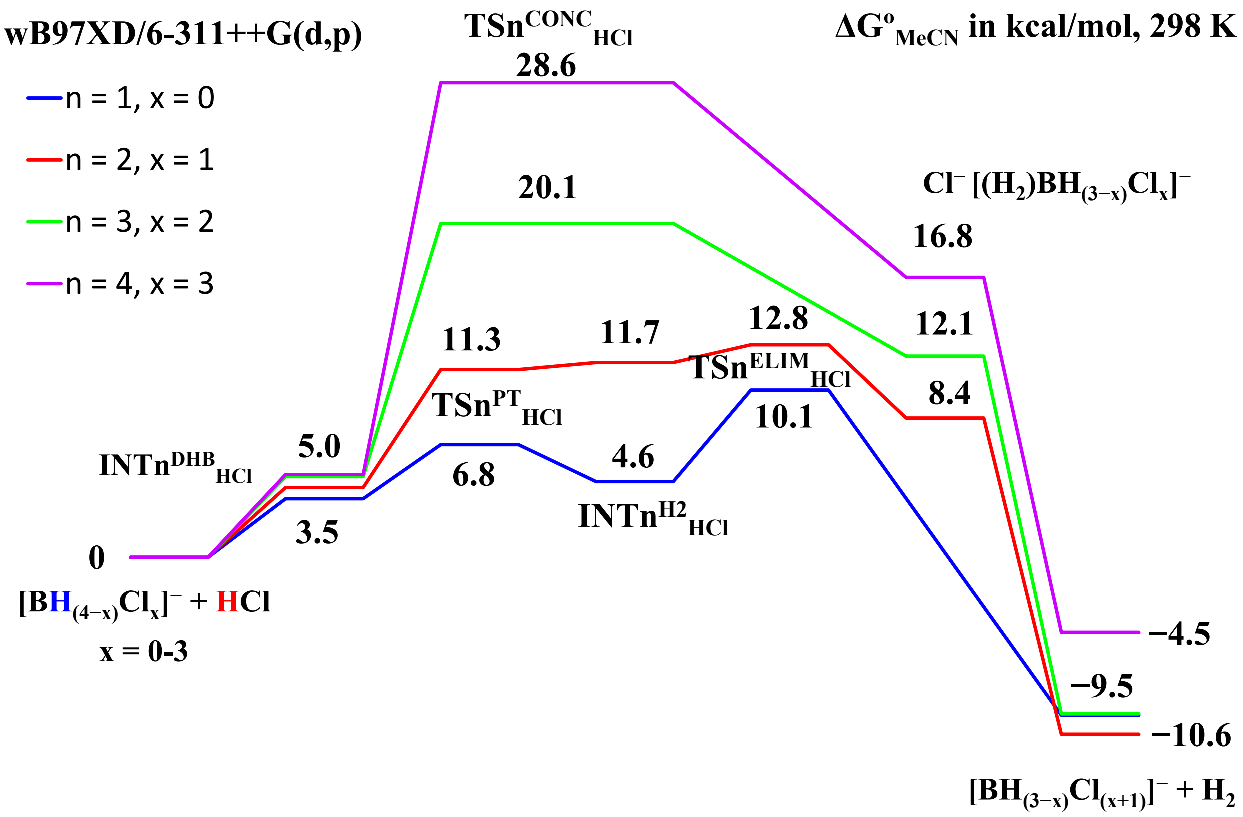

Figure S4. M06-calculated energy profile ($\Delta G°_{MeCN}$ in kcal/mol) of the reaction of BH_4^- with HBr in MeCN.

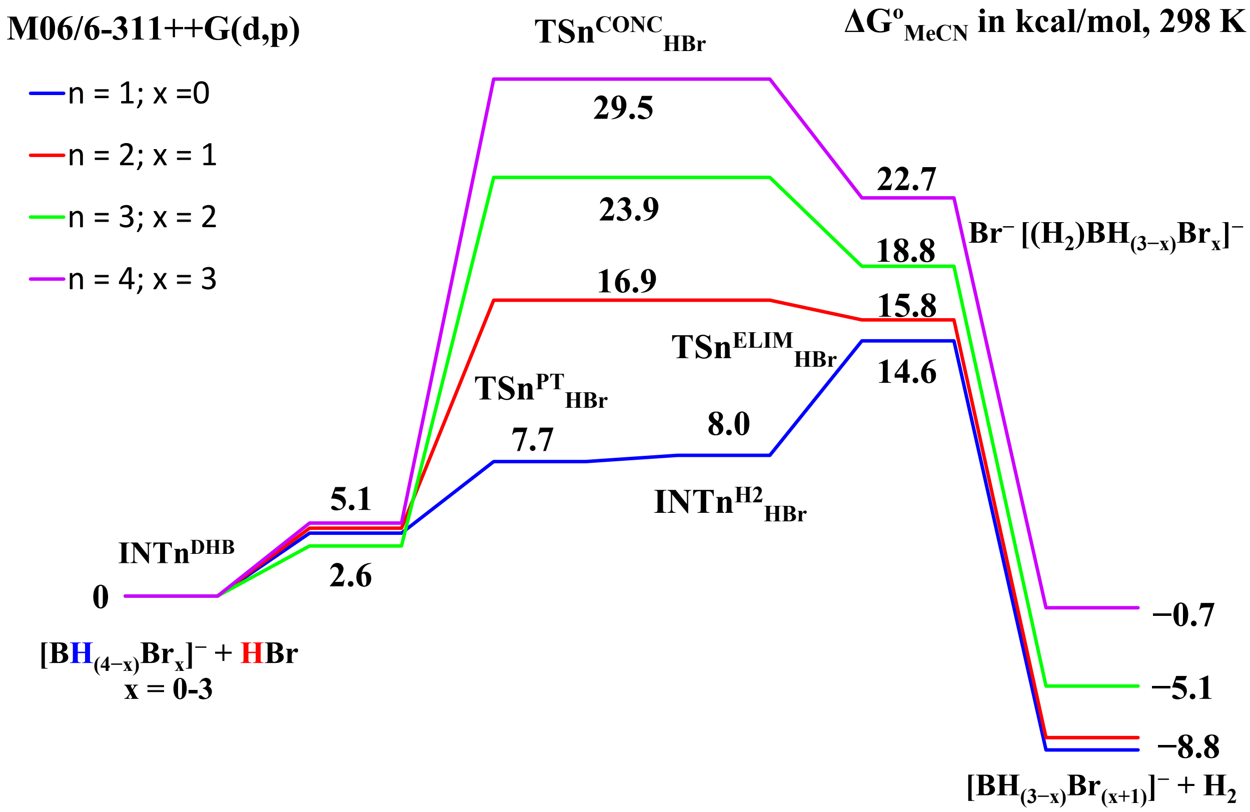

Figure S5. wB97XD-calculated energy profile ($\Delta G°_{MeCN}$ in kcal/mol) of the reaction of BH_4^- with HBr in MeCN.

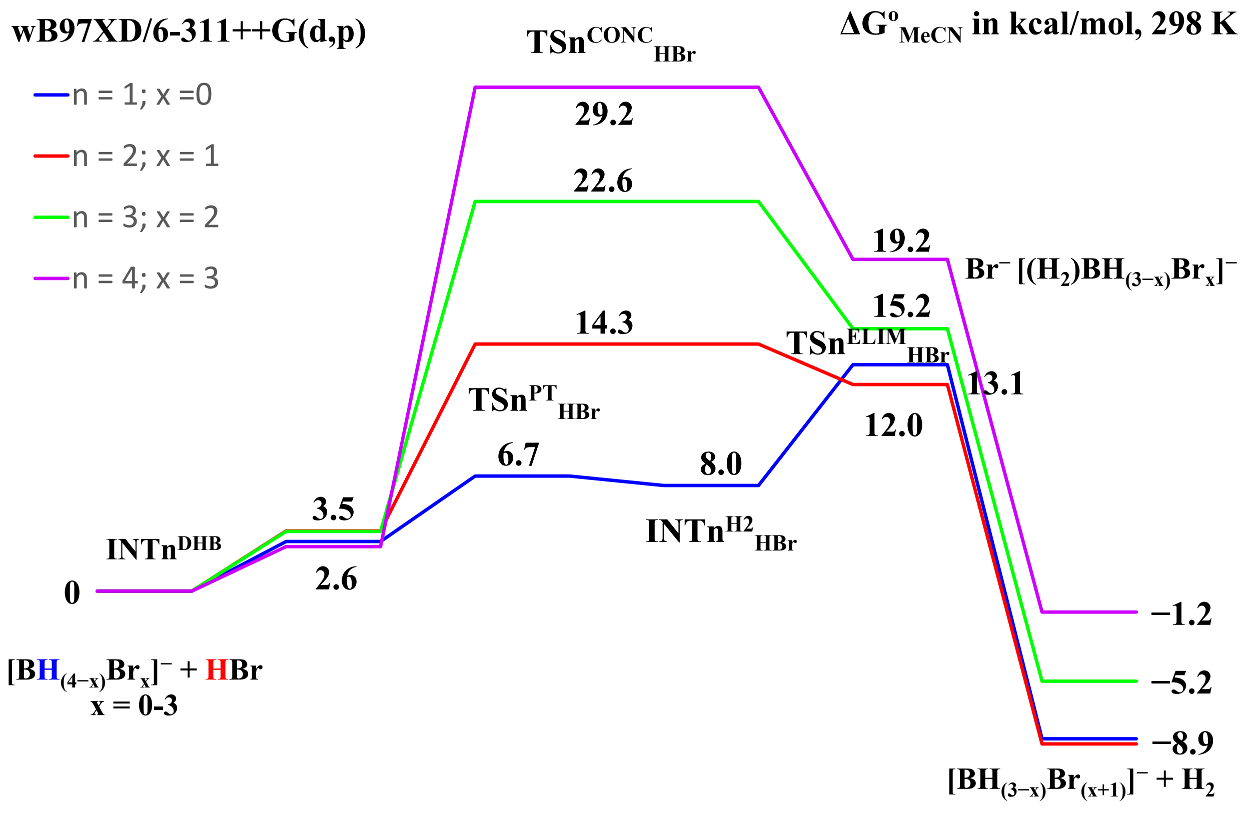

Table S5. Free Gibbs activation energy in MeCN ($\Delta G°_{MeCN}{}^{\ddagger(span)}$ in kcal/mol) of the transition state of H_2 elimination (**TSn**$^{ELIM}{}_{HX}$) and concerted transition state of proton-hydride transfer/H_2 elimination (**TSn**$^{CONC}{}_{HX}$) for reaction of BH_4^- with HX (X = F, Cl, Br) and their M06-optimized geometric parameters (distances $r_{(X–Y)}$ in Å).

**M06 TS**	$\Delta G°_{MeCN}{}^{\ddagger\ (span)}$	$r_{(B\cdots H_2)}$	$r_{(H–H)}$	$r_{(H\cdots X)}$	$r_{(B\cdots X)}$
$TS1^{CONC}{}_{HF}$	30.1	2.452	0.760	2.030	2.969
$TS2^{CONC}{}_{HF}$	22.4	1.940	0.781	1.818	3.335
$TS3^{CONC}{}_{HF}$	22.0	1.895	0.806	1.595	3.304
$TS4^{CONC}{}_{HF}$	28.9	1.926	0.799	1.627	3.306
$TS1^{ELIM}{}_{HCl}$	9.1	2.631	0.755	2.661	3.528
$TS2^{ELIM}{}_{HCl}$	12.9	1.861	0.773	2.460	3.945
$TS3^{CON}{}_{HCl}$	19.2	1.825	0.789	2.195	3.913
$TS4^{CONC}{}_{HCl}$	26.1	1.902	0.796	2.086	3.956
$TS1^{ELIM}{}_{HBr}$	14.6	2.528	0.758	2.741	3.725
$TS2^{CONC}{}_{HBr}$	16.9	2.004	0.772	2.563	4.231
$TS3^{CONC}{}_{HBr}$	23.9	2.008	0.789	2.303	4.282
$TS4^{CONC}{}_{HBr}$	29.5	1.837	0.795	2.226	4.355

Table S6. Free Gibbs activation energy in MeCN ($\Delta G°_{MeCN}{}^{\ddagger(span)}$ in kcal/mol) of the transition states of H_2 elimination (**TSn**$^{ELIM}{}_{HX}$) and concerted transition state of proton-hydride transfer/H_2 elimination (**TSn**$^{CONC}{}_{HX}$) for reaction of BH_4^- with HX (X = F, Cl, Br) and their wB97XD-optimized geometric parameters (distances $r_{(X–Y)}$ in Å).

**wB97XD TS**	$\Delta G°_{MeCN}{}^{\ddagger(span)}$	$r_{(B\cdots H_2)}$	$r_{(H–H)}$	$r_{(H\cdots X)}$	$r_{(B\cdots X)}$
$TS1^{CONC}{}_{HF}$	32.3	2.647	0.756	2.079	3.164
$TS2^{CONC}{}_{HF}$	23.1	1.993	0.773	1.864	3.500
$TS3^{CONC}{}_{HF}$	22.9	1.870	0.798	1.611	3.326
$TS4^{CONC}{}_{HF}$	31.1	1.921	0.789	1.665	3.350
$TS1^{ELIM}{}_{HCl}$	10.1	2.882	0.749	2.845	3.784
$TS2^{ELIM}{}_{HCl}$	12.8	1.933	0.763	2.641	4.140
$TS3^{CONC}{}_{HCl}$	20.1	1.798	0.776	2.327	4.020
$TS4^{CONC}{}_{HCl}$	28.6	1.859	0.785	2.147	3.960
$TS1^{ELIM}{}_{HBr}$	13.1	2.684	0.752	2.834	3.935
$TS2^{CONC}{}_{HBr}$	14.3	2.049	0.763	2.682	4.299
$TS3^{CONC}{}_{HBr}$	22.6	1.955	0.777	2.391	4.295
$TS4^{CONC}{}_{HBr}$	29.2	2.042	0.786	2.257	4.355

Table S7. Free Gibbs activation energy in dichloromethane ($\Delta G°_{DCM}^{‡}$ in kcal/mol)[5] of the concerted transition state of proton-hydride transfer/H_2 elimination (**TS^{CONC}_{ROH}**) for reaction of BH_4^- with HOR [ROH = MeOH, CF_3CH_2OH (TFE), $(CF_3)_2CHOH$ (HFIP)] and their M06-optimized geometric parameters.

Name of TS	$\Delta G°_{DCM}^{‡}$	$r_{(B\cdots H2)}$	$r_{(H–H)}$	$r_{(H\cdots O)}$	$r_{(B\cdots O)}$
$TS1^{CONC}_{MeOH}$	53.3	2.125	0.828	1.565	2.646
$TS2^{CONC}_{MeOH}$	30.1	2.335	0.810	1.692	3.539
$TS3^{CONC}_{MeOH}$	21.9	2.134	0.883	1.400	3.462
$TS4^{CONC}_{MeOH}$	18.8	2.260	0.930	1.312	3.541
$TS1^{CONC}_{TFE}$	44.5	2.183	0.789	1.788	2.800
$TS2^{CONC}_{TFE}$	26.1	2.012	0.815	1.652	3.240
$TS3^{CONC}_{TFE}$	24.3	1.951	0.863	1.436	3.268
$TS4^{CONC}_{TFE}$	21.6	2.026	0.899	1.349	3.258
$TS1^{CONC}_{HFIP}$	35.1	2.233	0.744	1.924	2.847
$TS2^{CONC}_{HFIP}$	21.3	2.291	0.788	1.799	3.246
$TS3^{CONC}_{HFIP}$	16.7[a]	1.912	0.859	1.440	3.270

[a] This value was calculated for the model reaction of $[((CF_3)_2CHO)((CF_3)(CH_3)CHO)BH_2]^-$ with $(CF_3)_2CHOH$ (HFIP)

Table S8. Hydride (HDA_{MeCN} in kcal/mol) and proton (PDA_{MeCN} in kcal/mol) donor abilities for $[B_6H_{(6-x)}Cl_x]^{2-}$ (x = 0–5) in MeCN.

	**Molecule**	**HDA_{MeCN}**	**PDA_{MeCN}**
	$[B_6H_6]^{2-}$	71.94	106.58
[1]	$[B_6ClH_5]^{2-}$	BH_{eq} 76.55 BH_{ax} 80.01	BH_{eq} 103.11 BH_{ax} 102.41
[1,2] (*cis*)	$[B_6Cl_2H_4]^{2-}$	BH_{cis} 80.97 BH_{trans} 84.67	BH_{cis} 100.42 BH_{trans} 99.70
[1,6] (*trans*)	$[B_6Cl_2H_4]^{2-}$	81.06	100.05
[1,2,3] (*fac*)	$[B_6Cl_3H_3]^{2-}$	88.60	96.64
[1,6,2] (*mer*)	$[B_6Cl_3H_3]^{2-}$	BH_{cis} 84.89 BH_{trans} 88.63	BH_{cis} 97.23 BH_{trans} 96.42
[1,2,3,4] (*cis*)	$[B_6Cl_4H_2]^{2-}$	92.54	95.67
[1,6,2,4] (*trans*)	$[B_6Cl_4H_2]^{2-}$	88.43	91.71
[1,2,3,4,6]	$[B_6Cl_5H]^{2-}$	95.65	89.27

Table S9. M06-computed geometric (distances $r_{(X\text{–}Y)}$ in Å, angles in degrees) parameters of hydrogen-bonded complexes of $[B_6H_{(6\text{–}x)}Cl_x]^{2–}$ (n = 0–5) with HCl (**INT^HB**), their energy formation energy ($\Delta G_f^{\circ MeCN}$ and $\Delta H_f^{\circ MeCN}$ in kcal/mol), electron densities at the H···B(B_2) bond (+3;−1) critical point (ϱ_c in au), Laplacian of electron density at critical point ($\nabla^2\varrho_c$ in au), bond ellipticity (ε) and energy of H···B(B_2) interaction ($E_{H\cdots B(B2)} = 0.5\cdot V(r)$ [1, 2] in kcal/mol) and donation energy (E^2_{HB} in kcal/mol) from σ(B–B–B) to σ*(H–Cl) estimated from 2nd-order perturbative analysis of donor acceptor interactions within natural-bond orbital (NBO) analysis.

N	INT^HB3	TS^PT	TS^ISO fac-apx	TS^ISO fac-fac*	$r_{(H_X\cdots B_3)}$	$r_{(XH)}$	$\Delta r_{(XH)}$	$\angle XH\cdots B_3$	$\Delta G_f^{\circ}{}_{MeCN}$	$\Delta H_f^{\circ}{}_{MeCN}$	ρ_c	$\nabla^2\rho_c$	ε	$E_{H\cdots B(B_2)}$	E^2_{HB}
	$[B_6H_6]^{2–}\cdots HCl$	0.7	20.1	17.3	2.006	1.339	0.045	180	1.8	−6.9	0.026	0.046	0.01	−3.7	9.77
[1]	$[B_6ClH_5]^{2–}\cdots HCl$	1.3/2.3	19.8/19.0	14.7/14.2	2.021	1.336	0.042	179	1.9	−6.7	0.026	0.046	0.03	−3.6	9.29
[1,2] (*cis*)	$B_6Cl_2H_4]^{2–}\cdots HCl$	2.1/2.2	19.4/17.9	11.6	2.037	1.333	0.039	179	2.1	−6.4	0.025	0.046	0.01	−3.4	9.14
[1,6] (*trans*)	$[B_6Cl_2H_4]^{2–}\cdots HCl$	3.6	16.7	14.2	2.112	1.326	0.032	179	2.6	−6.0	0.022	0.044	0.30	−3.0	8.52
[1,2,3] (*fac*)	$[B_6Cl_3H_3]^{2–}\cdots HCl$	2.1	17.4	13.3	2.058	1.330	0.036	179	2.2	−6.1	0.024	0.045	0.04	−3.2	8.81
[1,6,2] (*mer*)	$[B_6Cl_3H_3]^{2–}\cdots HCl$	3.9/4.2	16.0/15.0	10.6	2.121	1.325	0.031	178	2.6	−5.9	0.022	0.044	0.32	−3.0	8.41
[1,2,3,4] (*cis*)	$[B_6Cl_4H_2]^{2–}\cdots HCl$	4.8	13.8	10.4	2.133	1.323	0.029	178	2.7	−5.8	0.021	0.043	0.33	−2.9	8.23
[1,6,2,4] (*trans*)	$[B_6Cl_4H_2]^{2–}\cdots HCl$	7.2	–	9.5	2.235	1.316	0.022	179	2.7	−5.5	0.018	0.040	0.55	−2.4	7.56
[1,2,3,4,6]	$[B_6Cl_5H]^{2–}\cdots HCl$	8.0	–	7.9	2.234	1.315	0.021	178	2.7	−5.5	0.018	0.039	0.52	−2.4	7.49

Table S10. M06-computed geometric (distances $r_{(X\text{–}Y)}$ in Å, angles in degrees) parameters of hydrogen bonded complexes of $Cl^-\cdot[^{fac}HB_6H_{(6\text{–}x)}Cl_x]^-$ (x = 0–5) with HCl, their energy formation energy ($\Delta G_f^{\circ MeCN}$ and $\Delta H_f^{\circ MeCN}$ in kcal/mol), electron densities at the H···B(B_2) bond (+3;−1) critical point (ϱ_c in au), Laplacian of electron density at critical point ($\nabla^2\varrho_c$ in au), bond ellipticity (ε) and energy of H···B(B2) interaction ($E_{H\cdots B(B2)} = 0.5\cdot V(r)$[1, 2] in kcal/mol).

N	HB3 complex	$TS^{PT}{}_{2nd}$	$TS^{PT}{}_{2nd(rev)}$	$r_{(H_X\cdots B_3)}$	$r_{(XH)}$	$\Delta r_{(XH)}$	$\angle XH\cdots B_3$	$\Delta G_f^{\circ}{}_{MeCN}$	$\Delta H_f^{\circ}{}_{MeCN}$	ρ_c	$\nabla^2\rho_c$	ε	$E_{H\cdots B(B_2)}$
	$Cl^-\cdot[^{fac}HB_6H_6]^-\cdots HCl$	9.5	3.8	2.122	1.314	0.390	180	3.9	−4.1	0.020	0.044	0.01	−2.7
[1] (*cis*)	$Cl^-\cdot[^{fac}HB_6ClH_5]^-\cdots HCl$	11.1	2.8	2.143	1.312	0.388	178	7.0	−1.2	0.019	0.043	0.03	−2.6
[1] (*trans*)	$Cl^-\cdot[^{fac}HB_6ClH_5]^-\cdots HCl$	13.3	2.3	2.219	1.308	0.384	179	4.2	−3.8	0.017	0.040	0.29	−2.3
[1,2] (*fac*)	$Cl^-\cdot[^{fac}HB_6Cl_2H_4]^{2–}\cdots HCl$	15.0	1.4	2.228	1.307	0.383	177	6.7	−3.6	0.017	0.039	0.34	−2.2
[1,2] (*mer*)	$Cl^-\cdot[^{fac}HB_6Cl_2H_4]^{2–}\cdots HCl$	14.6	1.1	2.235	1.307	0.383	176	6.9	−3.5	0.017	0.040	0.33	−2.2
[1,6] (*mer*)	$Cl^-\cdot[^{fac}HB_6Cl_2H_4]^{2–}\cdots HCl$	15.3	1.4	2.229	1.307	0.383	179	3.9	−3.6	0.017	0.039	0.25	−2.2
[1,2,3] (*cis*)	$Cl^-\cdot[^{fac}HB_6Cl_3H_3]^{2–}\cdots HCl$	18.9	0.0	2.362	1.303	0.379	174	6.7	−3.1	0.014	0.035	1.08	−1.9
[1,6,2] (*trans*)	$Cl^-\cdot[^{fac}HB_6Cl_3H_3]^{2–}\cdots HCl$	19.0	0.2	2.343	1.302	0.378	178	4.3	−3.2	0.014	0.034	0.57	−1.8
[1,2,3,4] (*cis*)	$Cl^-\cdot[^{fac}HB_6Cl_4H_2]^{2–}\cdots HCl$	22.8	0.3	2.589	1.297	0.373	177	5.3	−2.6	0.008	0.022	0.10	−1.1
[1,6,2,4] (*trans*)	$Cl^-\cdot[^{fac}HB_6Cl_4H_2]^{2–}\cdots HCl$	23.2	0.4	2.351	1.301	0.377	179	1.3	−6.2	0.014	0.034	0.55	−2.2
[1,2,3,4,6]	$Cl^-\cdot[^{fac}HB_6Cl_5H]^{2–}\cdots HCl$	23.0	0.0	2.745	1.296	0.372	175	5.3	−2.5	0.006	0.016	0.38	−0.8

Table S6. M06-computed geometric parameters (distances $r_{(X–Y)}$ in Å, angles in °) of H_2 complexes of $Cl^-[(H_2)B_6H_{(5-x)}Cl_x]^-$ (x = 0–5) (**INT^{H2}**), their energy formation energy ($\Delta G_f^{\circ MeCN}$ and $\Delta H_f^{\circ MeCN}$ in kcal/mol), electron densities in (+3;−1) donation energy (E^2 in kcal/mol) from σ(B–H/Cl) to σ*(H–H–B) (**E^2_1**), from σ(H–H–B) to σ*(B–H/Cl) (**E^2_2**) and from p(Cl) to σ*(H–H–B) (**E^2_3**) estimated from 2nd-order perturbative analysis of donor acceptor interactions within natural-bond orbital (NBO) analysis.

N	H2-complex	$r_{(H\cdots H)}$	$r_{(B\cdots H1)}$	$\Delta r_{(B\cdots H1)}$	$r_{(B\cdots H_2)}$	$r_{(B\cdots H2)}$	$\Delta r_{(B\cdots H2)}$	$r_{(H2\cdots X)}$	$\Delta r_{(H2\cdots X)}$	$\Delta G_f^{\circ}{}_{MeCN}$	$\Delta H_f^{\circ}{}_{MeCN}$	E^2_1	E^2_2	E^2_3	$E_{H2\cdots X}$	$E_{H\cdots H}$	$E_{B\cdots H}$
**1**	$Cl^-\cdot[(H_2)B_6H_5]^-$	0.910	1.269	0.069	1.222	1.338	0.138	2.206	0.912	11.2	3.1	15.7/6.6/5.9/4.8<br>4.5/4.3/3.7<br>3.1/2.1	2.0<br>1.9	19.5<br>1.9	−4.7	−56.9	−82.2
[1,**2** (*cis*)	$Cl^-\cdot[(H_2)B_6H_4Cl]^-$	0.914	1.267	0.069	1.221	1.340	0.142	2.186	0.892	12.8	4.8	10.8/8.1/7.9/5.5<br>4.3/3.4/1.3/1.2<br>1.1/1.0/0.9	2.4/2.3<br>2.0/0.5	20.6<br>2.0	−4.9	−56.1	−82.4
1.**6**<br>(*trans*)	$Cl^-\cdot[(H_2)B_6H_4Cl]^-$	0.921	1.260	0.063	1.210	1.328	0.131	2.203	0.909	11.9	3.7	16.8/5.5/5.1<br>4.5/4.1/3.4<br>2.9/2.0/1.6	8.6/5.6<br>4.2/3.8<br>1.0	20.2<br>1.9	−4.7	−56.9	−82.2
1,2,**3** (*fac*)	$Cl^-\cdot[(H_2)B_6H_3Cl_2]^-$	0.918	1.263	0.067	1.224	1.351	0.155	2.128	0.834	14.7	6.2	17.8/16.5/14.3/9.9/<br>4.7/4.5/1.9/1.7<br>1.4/0.7/0.6	4.8/3.0/2.9	26.7<br>3.1	−5.9	−55.3	−82.6
1,2,**6**<br>(*mer*)	$Cl^-\cdot[(H_2)B_6H_3Cl_2]^-$	0.926	1.259	0.064	1.209	1.330	0.135	2.180	0.886	13.8	5.3	18.5/11.1/6.4<br>6.0/3.9/0.4/0.3	5.2/1.6/0.5	21.8<br>2.1	−5.0	−55.0	−84.6
1,6,**2**<br>(*mer*)	$Cl^-\cdot[(H_2)B_6H_3Cl_2]^-$	0.919	1.263	0.067	1.222	1.347	0.151	2.142	0.848	14.7	6.2	13.8/7.2/4.9<br>4.4/3.9/3.8<br>3.7/2.6/1.9/<br>1.7/1.3	3.5/2.4<br>2.1/1.8	25.1<br>2.5	−5.7	−55.4	−82.7
1,2,3,**4**<br>(*cis*)	$Cl^-\cdot[(H_2)B_6H_2Cl_3]^-$	0.928	1.256	0.063	1.214	1.342	0.149	2.122	0.828	15.5	7.1	30.8/8.1/<br>6.3/5.6/4.6<br>4.1/3.8/3/7	5.3/3.1/1.1	26.5<br>2.6	−6.0	−54.3	−84.6
1,6,2,**3**<br>(*cis*)	$Cl^-\cdot[(H_2)B_6H_2Cl_3]^-$	0.923	1.259	0.065	1.228	1.362	0.168	2.073	0.779	15.3	6.9	15.6/7.0/6.1<br>5.2/5.0/2.0/1.4	4.1/3.7/1.6	33.2<br>3.3	−7.1	−54.3	−83.0
1,6,2,**4**<br>(*trans*)	$Cl^-\cdot[(H_2)B_6H_2Cl_3]^-$	0.931	1.256	0.062	1.211	1.337	0.143	2.140	0.846	16.2	7.6	18.1/6.2/5.5/3.9	3.2/1.7/1.6<br>1.3/1.0	24.9<br>2.5	−5.7	−54.2	−84.8
1,2,3,4,**6**	$Cl^-\cdot[(H_2)B_6HCl_4]^-$	0.935	1.252	0.060	1.218	1.355	0.163	2.064	0.770	16.9	8.4	22.1/21.0/20.1<br>19.4/17.1/12.6<br>10.1/9.7/8.3/7.5	–	40.2<br>30.1<br>5.35	−7.3	−53.1	−84.8

Figure S6. M06-optimized geometries of the protonated forms $[^{fac}HB_6H_{(6-x)}Cl_x]^-$ (x = 0–4) and their free Gibbs formation energies ($\Delta G_f^{\circ MeCN}$ in kcal/mol).

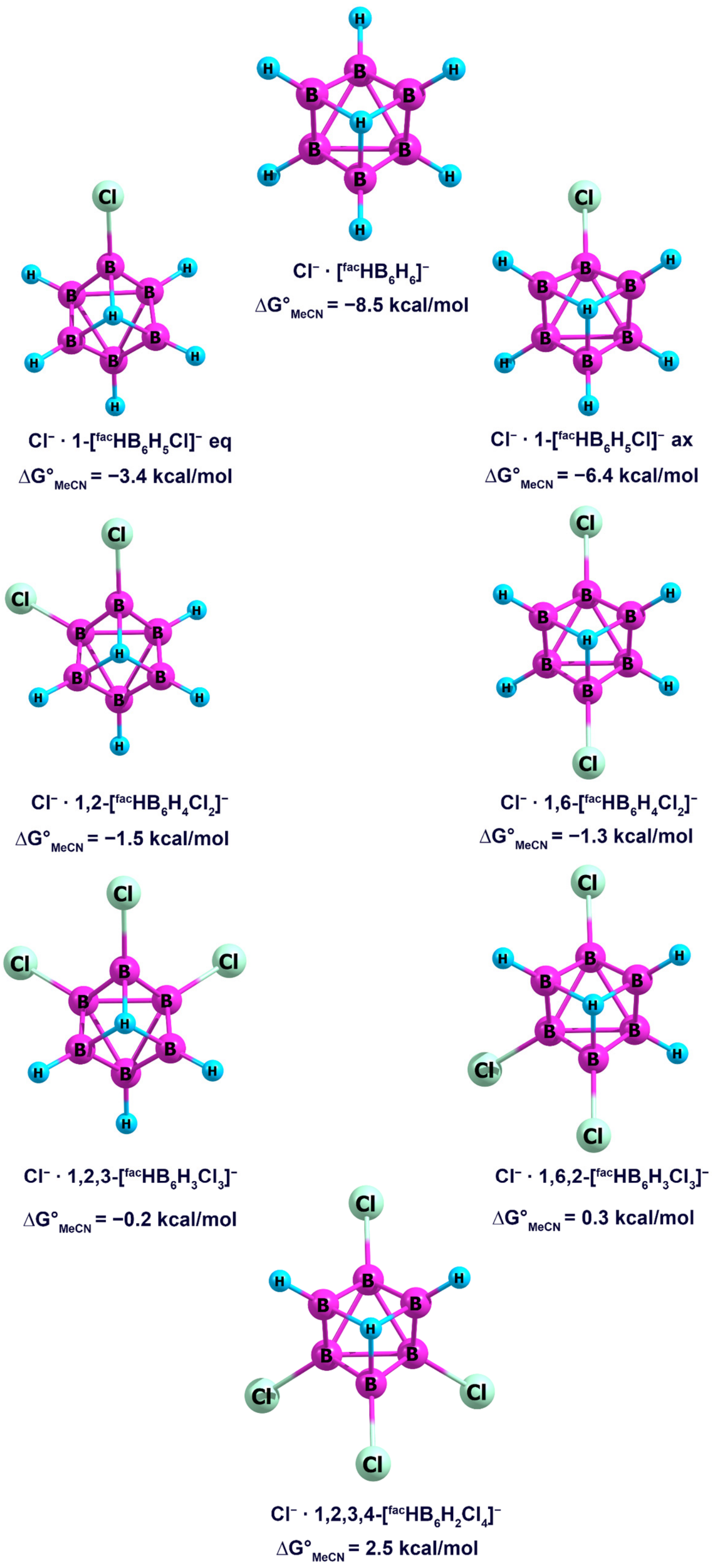

Figure S7. wB97XD-calculated energy profile of halogenation of $[B_6H_{(6-x)}Cl_x]^{2-}$ (x = 0–5) by HCl for the pathway: $[B_6H_6]^{2-}$ → 1-$[B_6H_5Cl]^{2-}$ → 1,2-$[B_6H_4Cl_2]^{2-}$ (*cis*) → 1,2,3-$[B_6H_3Cl_3]^{2-}$ (*fac*) → 1,2,3,4-$[B_6H_2Cl_4]^{2-}$ (*cis*) → 1,2,3,4,6-$[B_6HCl_5]^{2-}$.

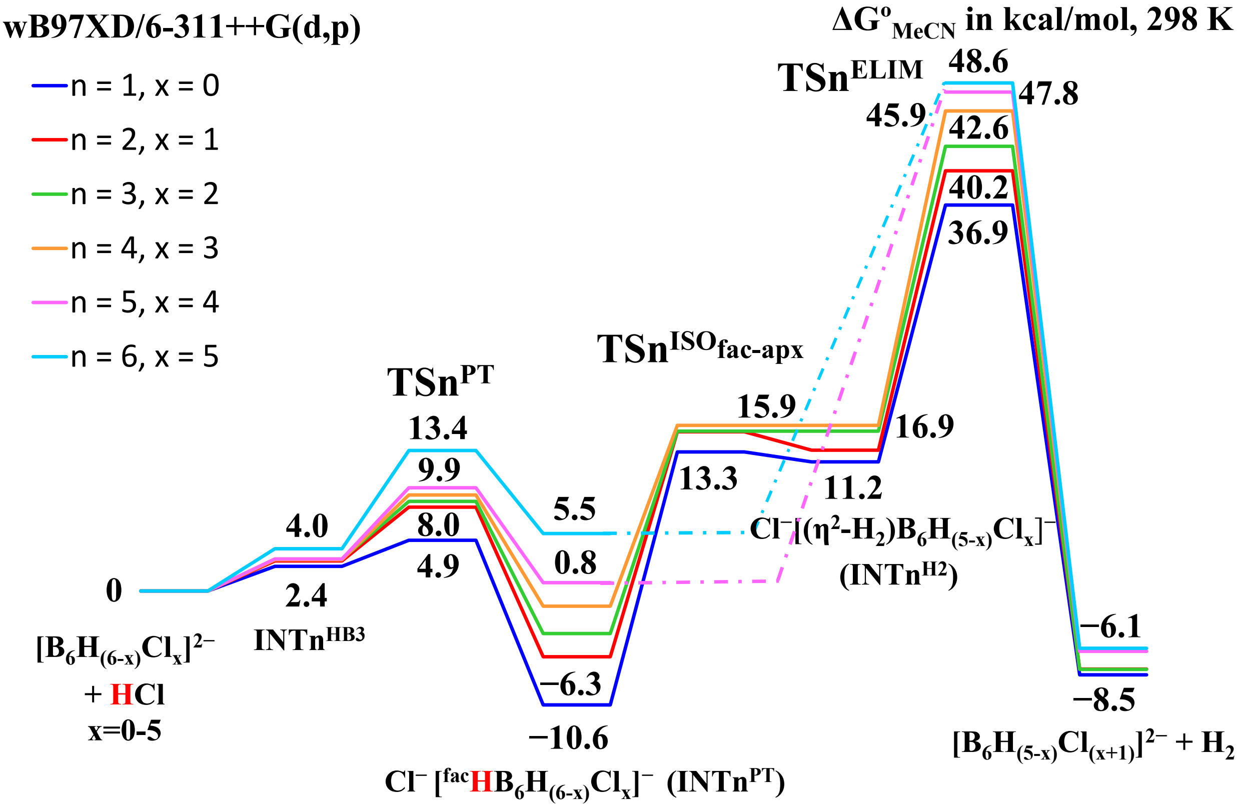

Table S12. Activation barriers ($\Delta G°_{MeCN}$‡, in kcal/mol) for TSs for the reaction of $[B_6H_6]^{2-}$ with HCl (pK_a^{MeCN}= 10.3)[42] in MeCN computed at the wB97XD/6-311++G(d,p) theory level.

n	N of position	$\Delta G°_{MeCN}$‡ (TSn^{PT}),a	$\Delta G°_{MeCN}$‡ ($TSn^{ISOfac-apx}$),b	$\Delta G°_{MeCN}$‡ (TSn^{ELIM}),c	$\Delta G°_{MeCN}$‡ (span),d
1	**1**	2.5	24.2	24.6	47.8
2	1,**2** (*cis*)	3.1	21.9	29.5	46.5
	1,**6** (*trans*)	5.1	21.5	26.7	51.2
3	1,2,**3** (*fac*)	5.5	19.4	27.2	46.6
	1,2,**6** (*mer*)	5.5	19.1	30.1	48.3
	1,6,**2** (*mer*)	6.4	19.7	28.0	47.7
4	1,2,3,**4** (*cis*)	6.2	17.3	30.1	47.4
	1,6,2,**3** (*cis*)	6.0	16.9	30.5	47.4
	1,6,2,**4** (*trans*)	6.0	41.8*		46.4
5	1,2,3,4,**6**	6.8	44.7*		47.8
	1,6,2,4,**3**	9.2	42.3*		46.0
6	1,2,3,4,6,**5**	9.4	44.6*		48.6

a Calculated as $\Delta G°_{MeCN}$‡ (**TSn^{PT}**) = $G°_{MeCN}$(**TSn^{PT}**) - $G°_{MeCN}$(**$INTn^{HB3}$**) b Calculated as $\Delta G°_{MeCN}$‡ (**$TSn^{ISOfac-apx}$**) = $G°_{MeCN}$(**$TSn^{ISOfac-apx}$**) - $G°_{MeCN}$(**$INTn^{PT}$**); c Calculated as $\Delta G°_{MeCN}$‡(**TSn^{ELIM}**) = $G°_{MeCN}$(**TSn^{ELIM}**) - $G°_{MeCN}$(**$INTn^{H2}$**); d Calculated as $\Delta G°_{MeCN}$‡(span) = $G°_{MeCN}$(**TSn^{ELIM}**) - $G°_{MeCN}$(**$INTn^{H2}$**) for n=1–4 and as $\Delta G°_{MeCN}$‡ (span) = $G°_{MeCN}$(**TSn^{ELIM}**) - $G°_{MeCN}$($[B_6H_{(6-x)}Cl_x]^{2-}$) - $G°_{MeCN}$(HCl) for n=5–6; * **TSn^{CONC}** for 5th and 6th halogenation steps.

References

1. Espinosa, E.; Alkorta, I.; Rozas, I.; Elguero, J.; Molins, E., About the evaluation of the local kinetic, potential and total energy densities in closed-shell interactions. *Chem. Phys. Lett.* **2001,** 336, (5–6), 457-461.
2. Espinosa, E.; Molins, E.; Lecomte, C., Hydrogen bond strengths revealed by topological analyses of experimentally observed electron densities. *Chem. Phys. Lett.* **1998,** 285, (3–4), 170-173.
3. Grant, D. J.; Dixon, D. A.; Camaioni, D.; Potter, R. G.; Christe, K. O., Lewis Acidities and Hydride, Fluoride, and X^- Affinities of the $BH_{3-n}X_n$ Compounds for (X = F, Cl, Br, I, NH_2, OH, and SH) from Coupled Cluster Theory. *Inorg. Chem.* **2009,** 48, (18), 8811-8821.
4. Golub, I. E.; Filippov, O. A.; Belkova, N. V.; Epstein, L. M.; Shubina, E. S., Hydride donating abilities of the tetracoordinated boron hydrides. *J. Organomet. Chem.* **2018,** 865, 247-256.
5. Golub, I. E.; Filippov, O. A.; Gulyaeva, E. S.; Gutsul, E. I.; Belkova, N. V., The Interplay of Proton Accepting and Hydride Donor Abilities in the Mechanism of Step-Wise Boron Hydrides Alcoholysis. *Inorg. Chim. Acta* **2017,** 456, 113-119.
