# Supplementary figures and images for: The Reaction of Hydrogen Halides with Tetrahydroborate Anion and Hexahydro-closo-hexaborate Dianion
Source: Molecules. 2021 Jun 20;26(12):3754. doi: 10.3390/molecules26123754 (PMC8235096; doi:10.3390/molecules26123754)

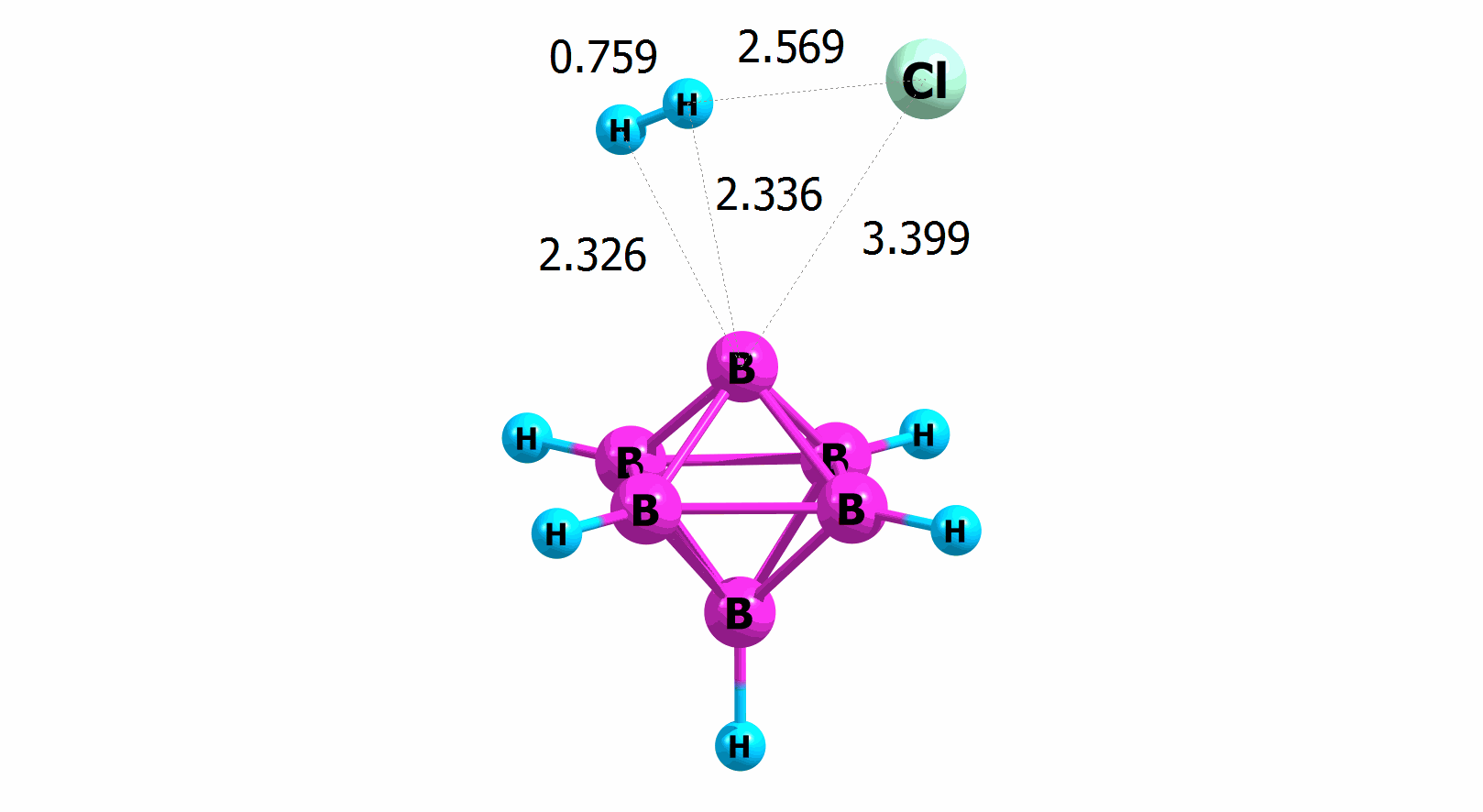

Supplement: Supplementary file 1 [file molecules-26-03754-s001.zip › molecules-1220861-supplementary/GIF/B6H6/B6H6_HCl_TS1_ELIM.gif]

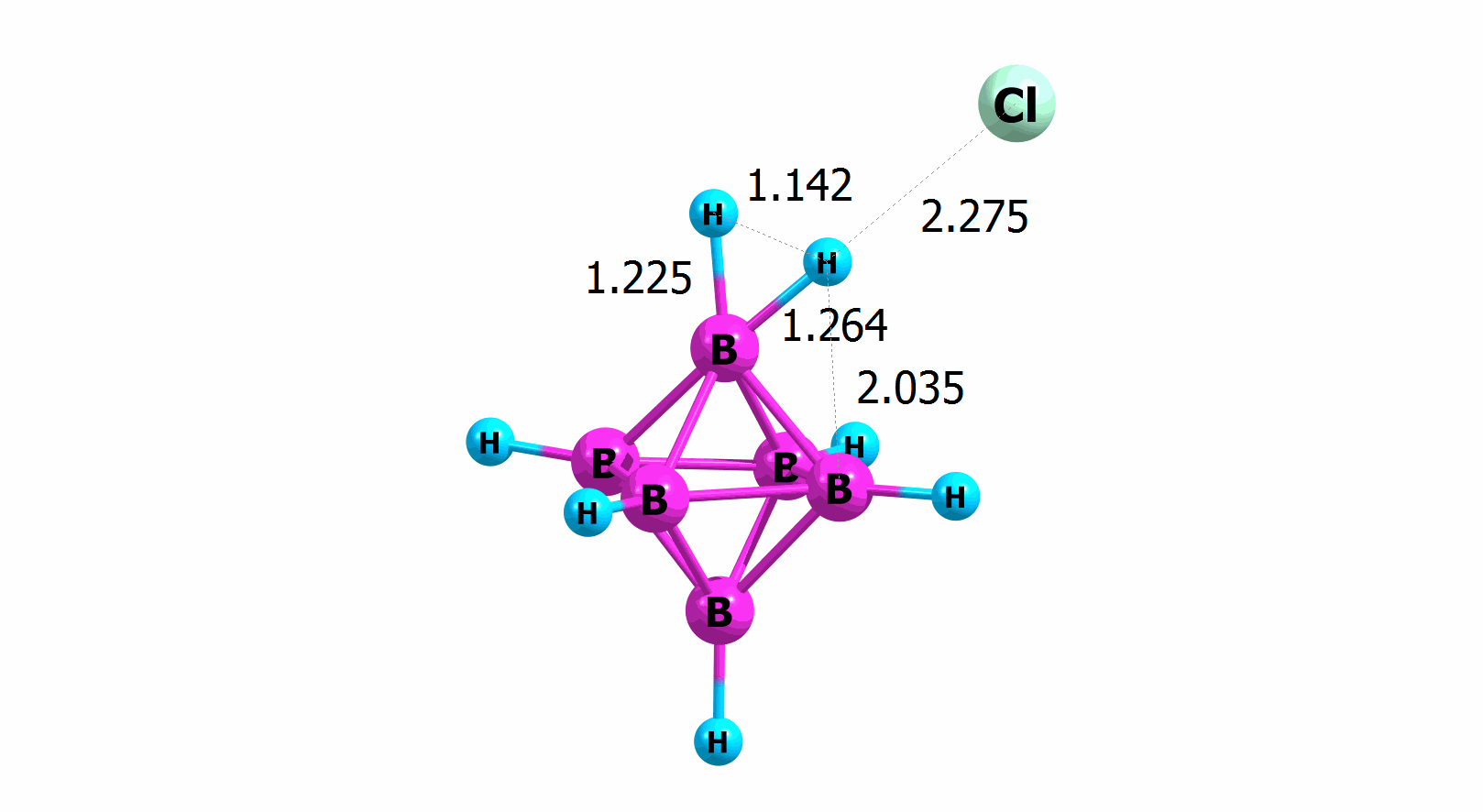

Supplement: Supplementary file 1 [file molecules-26-03754-s001.zip › molecules-1220861-supplementary/GIF/B6H6/B6H6_HCl_TS1_ISO_fac_apx.gif]

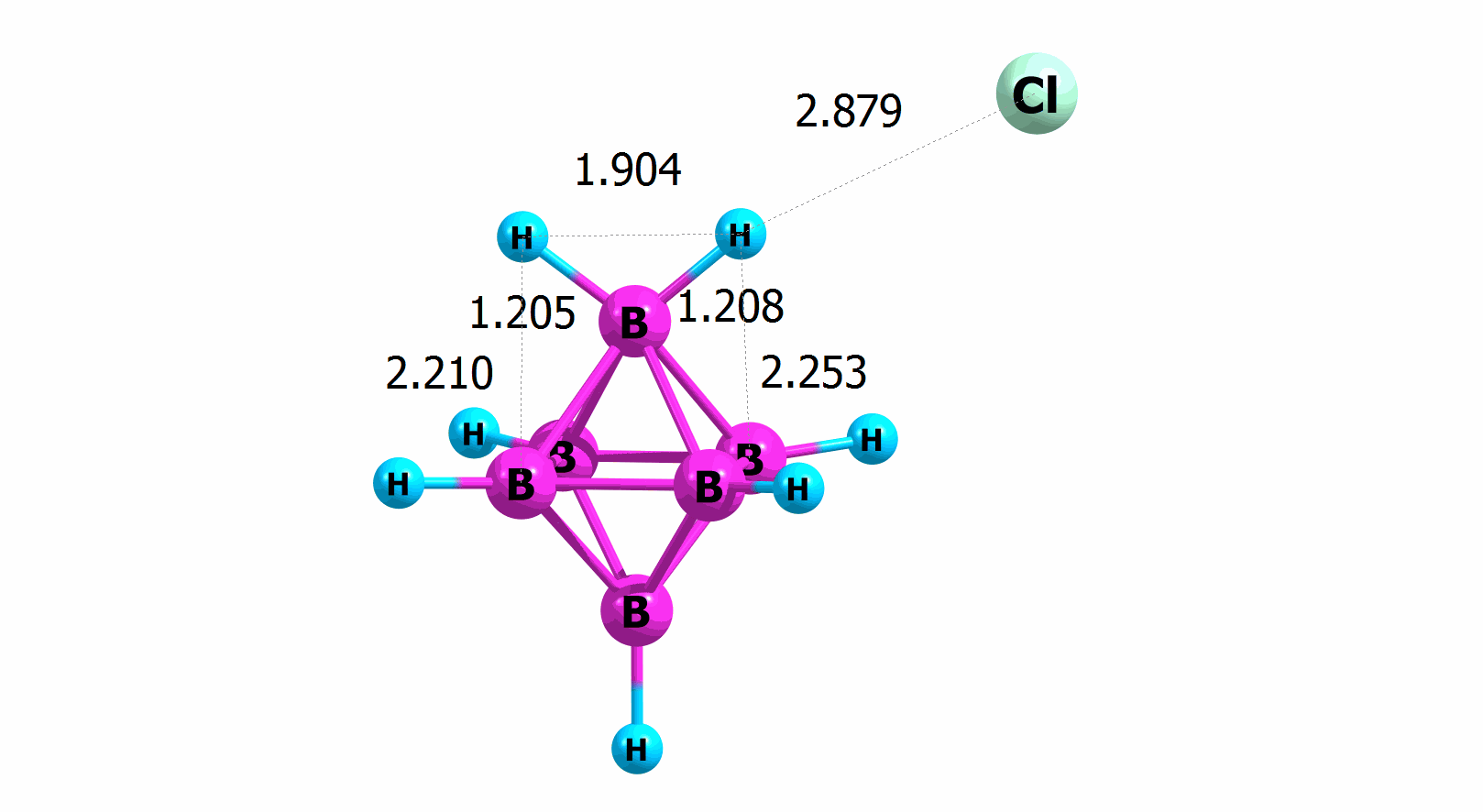

Supplement: Supplementary file 1 [file molecules-26-03754-s001.zip › molecules-1220861-supplementary/GIF/B6H6/B6H6_HCl_TS1_ISO_fac_fac.gif]

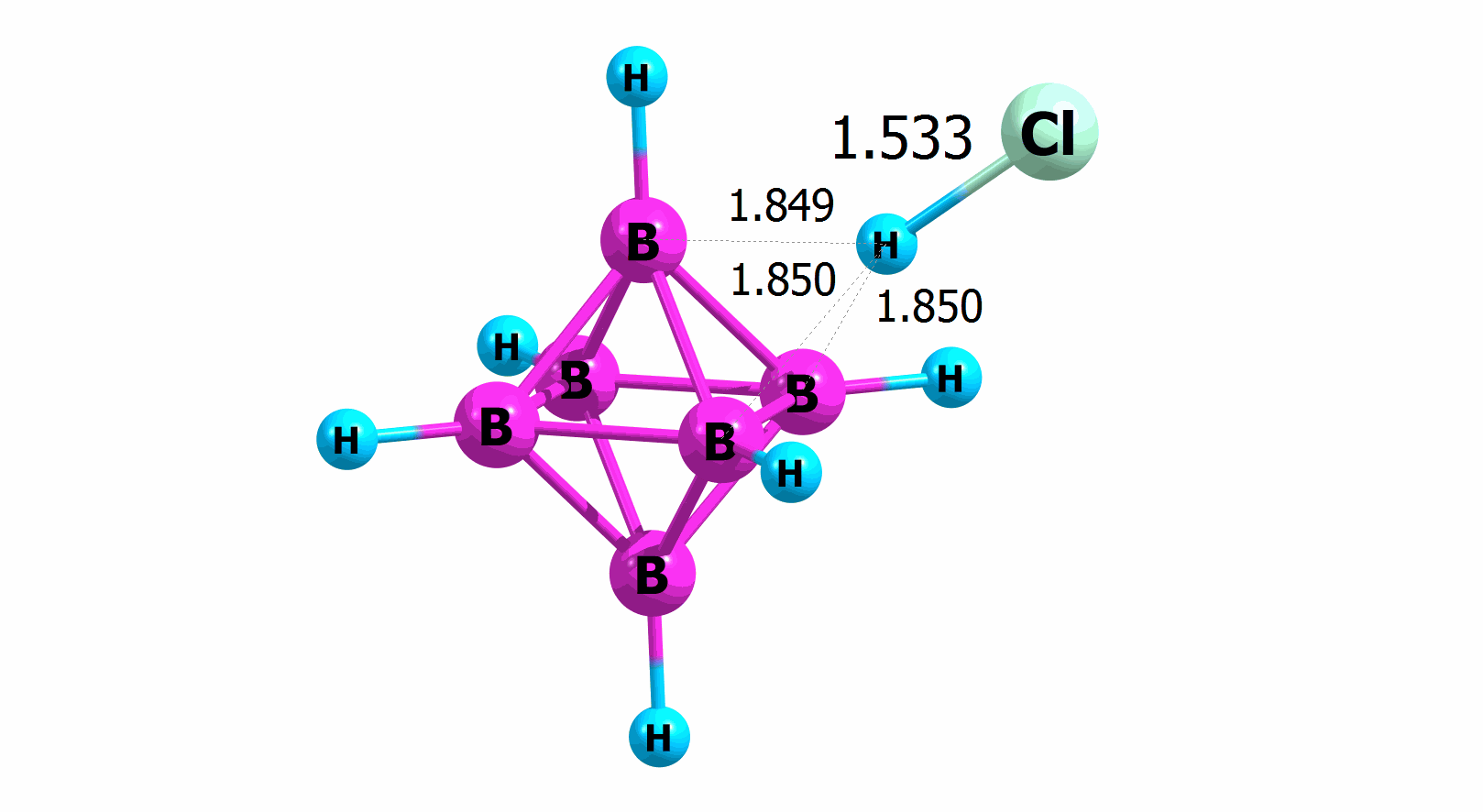

Supplement: Supplementary file 1 [file molecules-26-03754-s001.zip › molecules-1220861-supplementary/GIF/B6H6/B6H6_HCl_TS1_PT.gif]

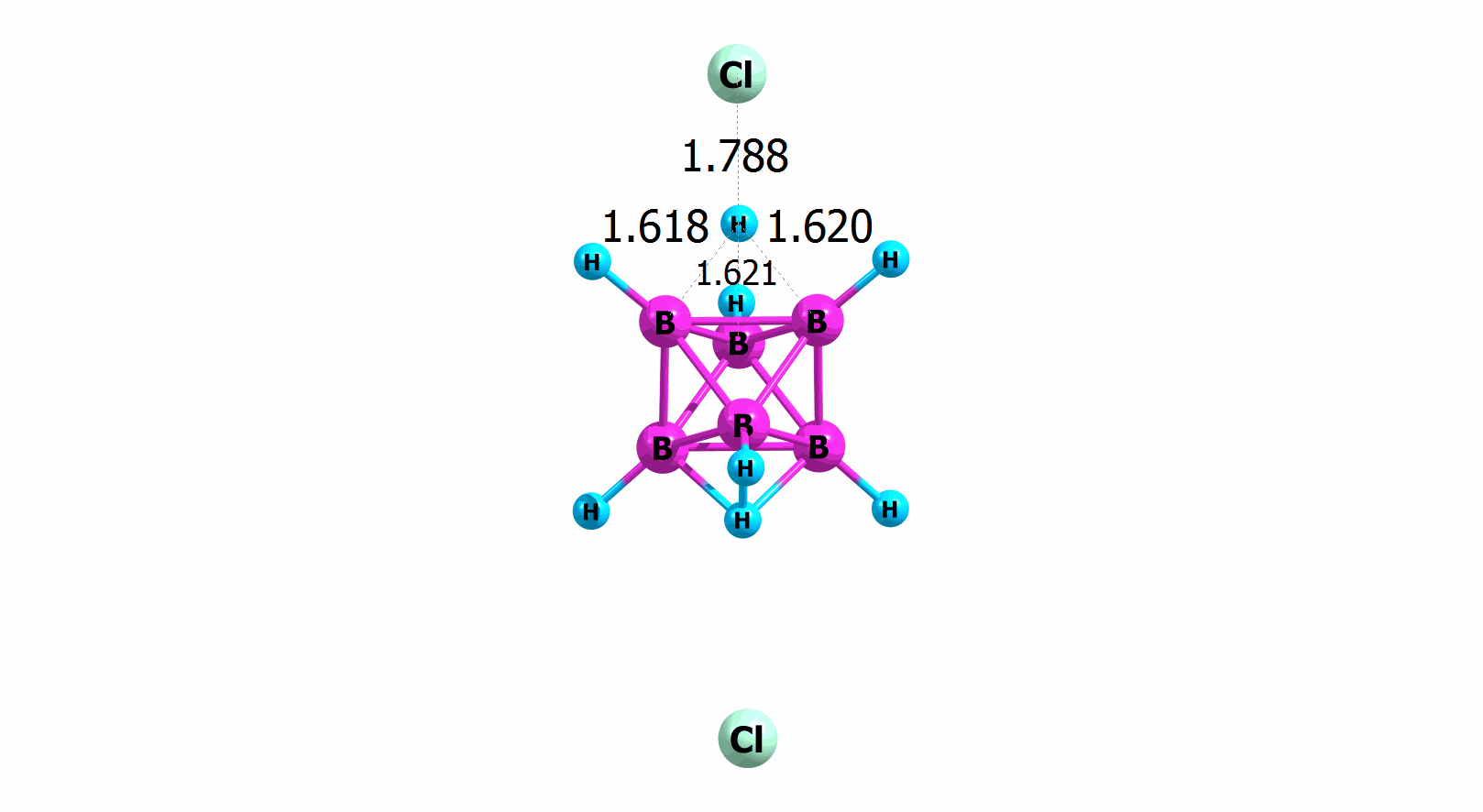

Supplement: Supplementary file 1 [file molecules-26-03754-s001.zip › molecules-1220861-supplementary/GIF/B6H6/B6H6_HCl_TS1_PT2nd.gif]

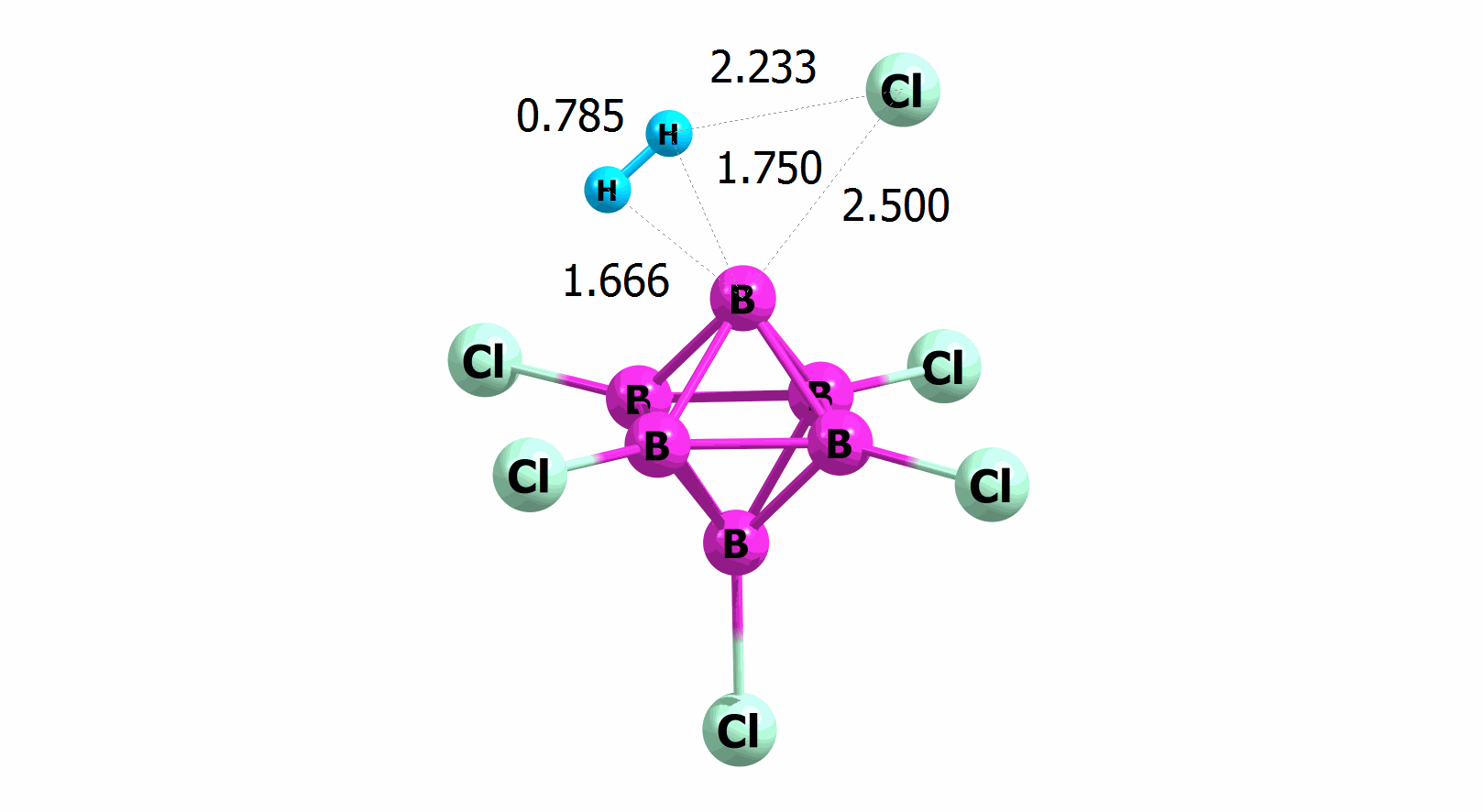

Supplement: Supplementary file 1 [file molecules-26-03754-s001.zip › molecules-1220861-supplementary/GIF/B6H6/B6H6_HCl_TS6_CONC.gif]

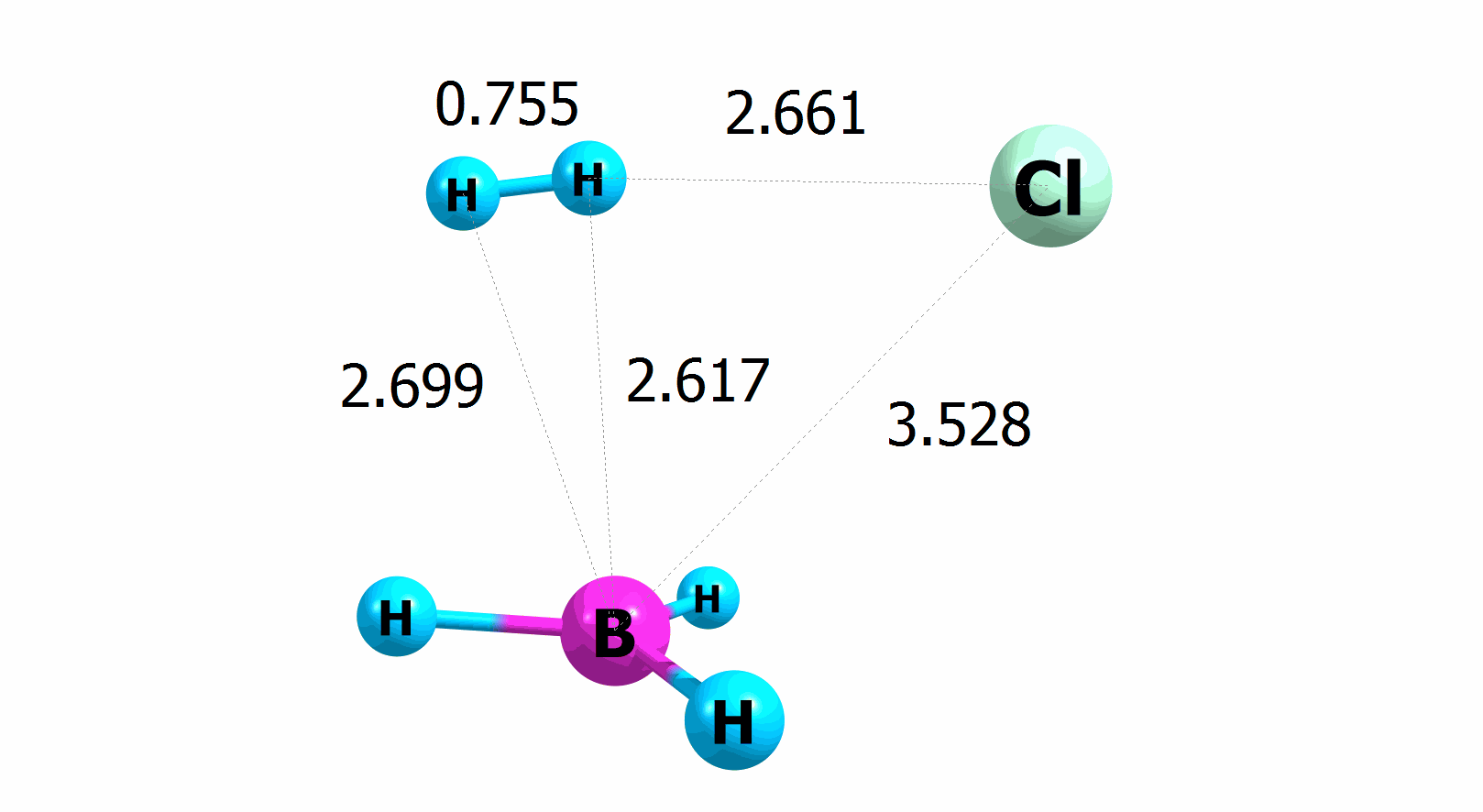

Supplement: Supplementary file 1 [file molecules-26-03754-s001.zip › molecules-1220861-supplementary/GIF/BH4/BH4_HCl_TS1_ELIM.gif]

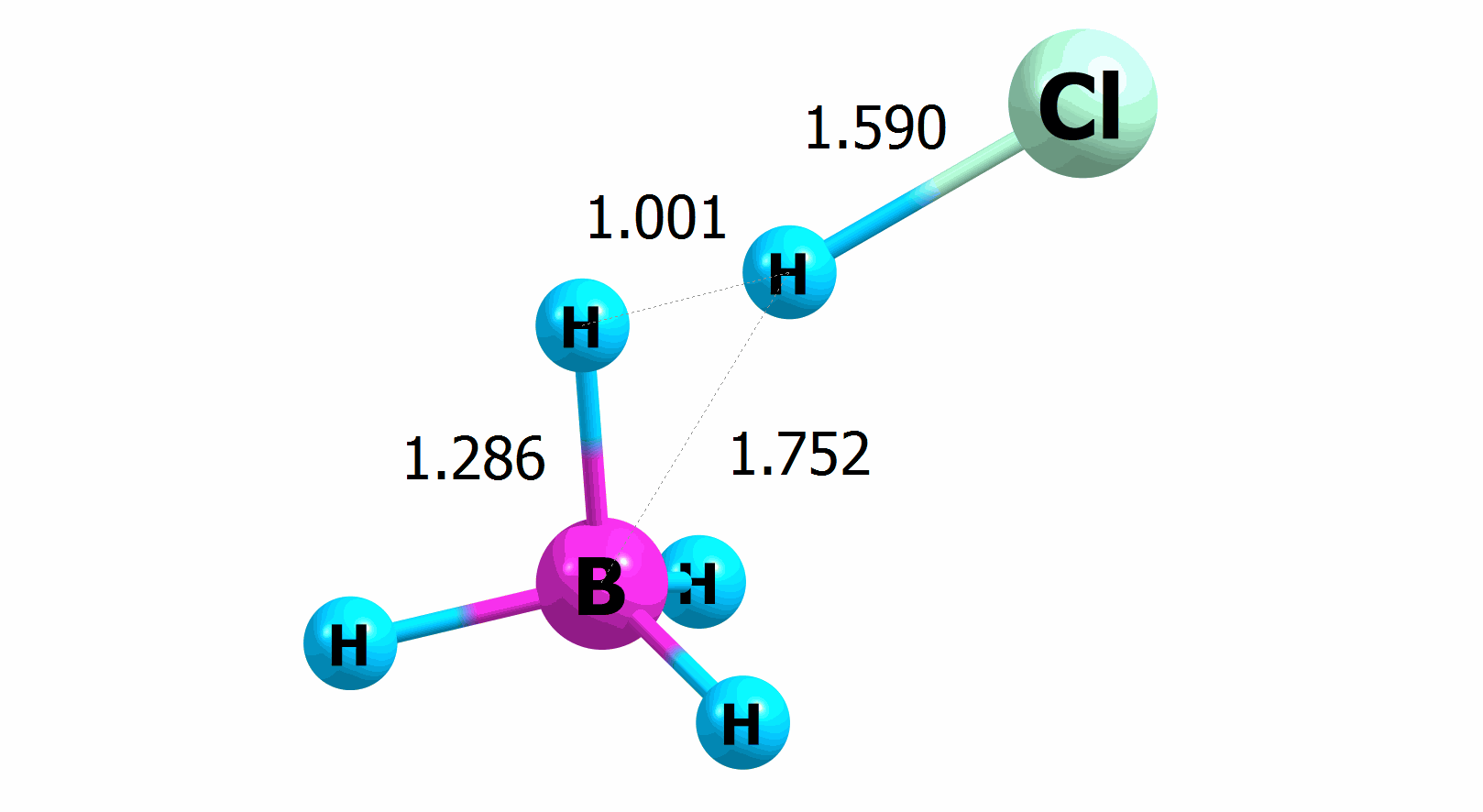

Supplement: Supplementary file 1 [file molecules-26-03754-s001.zip › molecules-1220861-supplementary/GIF/BH4/BH4_HCl_TS1_PT.gif]

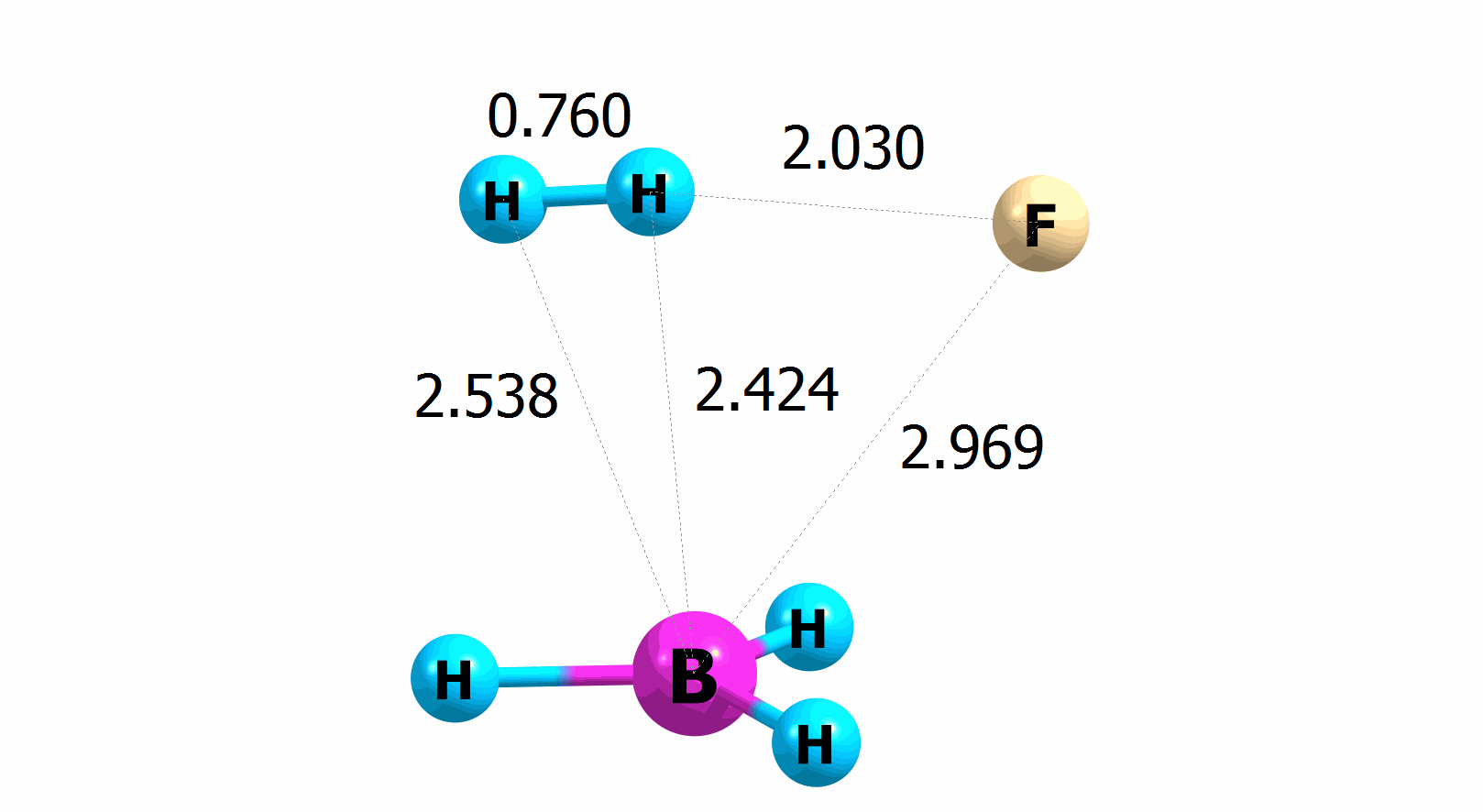

Supplement: Supplementary file 1 [file molecules-26-03754-s001.zip › molecules-1220861-supplementary/GIF/BH4/BH4_HF_TS_CONC.gif]
